# The Nordic Nutrition Recommendations 2022 – prioritisation of topics for *de novo* systematic reviews

**DOI:** 10.29219/fnr.v65.7828

**Published:** 2021-10-08

**Authors:** Anne Høyer, Jacob Juel Christensen, Erik Kristoffer Arnesen, Rikke Andersen, Hanna Eneroth, Maijaliisa Erkkola, Eva Warensjö Lemming, Helle Margrete Meltzer, Þórhallur Ingi Halldórsson, Inga Þórsdóttir, Ursula Schwab, Ellen Trolle, Rune Blomhoff

**Affiliations:** 1 The Norwegian Directorate of Health, Oslo, Norway; 2 Norwegian National Advisory Unit on Familial Hypercholesterolemia, Oslo University Hospital, Oslo, Norway; 3 Department of Nutrition, University of Oslo, Oslo, Norway; 4 National Food Institute, Technical University of Denmark (DTU), Kgs. Lyngby, Denmark; 5 The Swedish Food Agency, Uppsala, Sweden; 6 Department of Food and Nutrition, University of Helsinki, Helsinki, Finland; 7 Division of Infectious Diseases and Environmental Health, Norwegian Institute of Public Health, Oslo, Norway; 8 School of Health Sciences, University of Iceland, Reykjavík, Iceland; 9 School of Medicine, Institute of Public Health and Clinical Nutrition, University of Eastern Finland, Kuopio Campus, Kuopio, Finland; 10 Department of Medicine, Endocrinology and Clinical Nutrition, Kuopio University Hospital, Kuopio, Finland; 11 Department of Clinical Service, Division of Cancer Medicine, Oslo University Hospital, Oslo, Norway

**Keywords:** dietary reference values, food-based dietary guidelines, systematic reviews, Nordic countries, the Baltics, national food and health authorities, evidence-based nutrition, nutrient recommendations

## Abstract

**Background:**

As part of the process of updating national dietary reference values (DRVs) and food-based dietary guidelines (FBDGs), the Nordic Nutrition Recommendations 2022 project (NNR2022) will select a limited number of topics for systematic reviews (SRs).

**Objective:**

To develop and transparently describe the results of a procedure for prioritisation of topics that may be submitted for SRs in the NNR2022 project.

**Design:**

In an open call, scientists, health professionals, national food and health authorities, food manufacturers, other stakeholders and the general population in the Nordic and Baltic countries were invited to suggest SR topics. The NNR2022 Committee developed scoping reviews (ScRs) for 51 nutrients and food groups aimed at identifying potential SR topics. These ScRs included the relevant nominations from the open call. SR topics were categorised, ranked and prioritised by the NNR2022 Committee in a modified Delphi process. Existing qualified SRs were identified to omit duplication.

**Results:**

A total of 45 nominations with suggestion for more than 200 exposure–outcome pairs were received in the public call. A number of additional topics were identified in ScRs. In order to omit duplication with recently qualified SRs, we defined criteria and identified 76 qualified SRs. The NNR2022 Committee subsequently shortlisted 52 PI/ECOTSS statements, none of which overlapped with the qualified SRs. The PI/ECOTSS statements were then graded ‘High’ (*n* = 21), ‘Medium’ (*n* = 9) or ‘Low’ (*n* = 22) importance, and the PI/ECOTSS statements with ‘High’ were ranked in a Delphi process. The nine top prioritised PI/ECOTSS included the following exposure–outcome pairs: 1) plant protein intake in children and body growth, 2) pulses/legumes intake, and cardiovascular disease and type 2 diabetes, 3) plant protein intake in adults, and atherosclerotic/cardiovascular disease and type 2 diabetes, 4) fat quality and mental health, 5) vitamin B^12^ and vitamin B^12^ status, 6) intake of white meat (no consumption vs. high consumption and white meat replaced with red meat), and all-cause mortality, type 2 diabetes and risk factors, 7) intake of n-3 LPUFAs from supplements during pregnancy, and asthma and allergies in the offspring, 8) nuts intake and cardiovascular disease (CVD) and type 2 diabetes in adults, 9) dietary fibre intake (high vs. low) in children and bowel function.

**Discussion:**

The selection of topics for *de novo* SRs is central in the NNR2022 project, as the results of these SRs may cause adjustment of existing DRVs and FBDGs. That is why we have developed this extensive process for the prioritisation of SR topics. For transparency, the results of the process are reported in this publication.

**Conclusion:**

The principles and methodologies developed in the NNR2022 project may serve as a framework for national health authorities or organisations when developing national DRVs and FBDGs. This collaboration between the food and health authorities in Denmark, Estonia, Finland, Iceland, Latvia, Lithuania, Norway and Sweden represents an international effort for harmonisation and sharing of resources and competence when developing national DRVs and FBDGs.

## Popular scientific summary

- Qualified systematic reviews will be the main foundation for revising dietary reference values and food-based dietary guidelines in the Nordic Nutrition Recommendation 2022.
- This paper describes the results of an open, transparent six-step procedure to identify topics that will be prioritised for *de novo* systematic reviews by the Nordic Nutrition Recommendation 2022 project.

Systematic reviews (SRs) (1) are the preferred method to summarise the current evidence on the causal relationship between nutrient- or food group exposure and a health outcome. Whilst several thousand SRs have been published in the field of diet and nutrition, only a limited number of SRs have adhered to the extensive principles and methodologies required to be identified as ‘qualified SRs’ (2–4) (see Step 3 later) by the Nordic Nutrition Recommendations 2022 (NNR2022) project. Qualified SRs will be the main foundation when the NNR2022 project revises national dietary reference values (DRVs) and food-based dietary guidelines (FBDGs) for the Nordic and Baltic countries. Production of qualified SRs is costly, and there are few dedicated, stable and long-term funding opportunities for the production of qualified SRs by any national food or health authorities, or international food and health organisation (5).

In the field of cancer, the World Cancer Research Fund International (WCRF) regularly produces qualified SRs on diet, obesity and physical activity and their causal relationship with the 17 most common cancers (6). The ‘Dietary Guidelines for Americans’ project (7), which is updated every 5 years, and the joint US-Canadian ‘Dietary Reference Intakes’ project (8) organised by The National Academy of Sciences, Engineering and Medicine also produce qualified SRs for the selected exposure–health outcome pairs. Some additional national food and health authorities or international food and health organisations also produce a limited number of qualified SRs. These are precious and authoritative sources for national health authorities developing DRVs and FBDGs.

In the NNR2022 project, we have considered multiple health outcomes from 51 nutrient or food group exposures, representing in total several hundred possible exposure–health outcome pairs. Thus, the available qualified SRs from national food and health authorities and international food and health organisations cover only a subset of all possible nutrient/food group relationships with the main outcomes considered when setting DRVs and FBDGs in the NNR2022 project. The NNR2022 project plans to use the available budget to develop a limited set (i.e. 9) of *de novo* SRs, which adhere to the extensive principles and methodologies for qualified SRs.

National authorities have most often used an *ad hoc* procedure when prioritising topics for SRs. Recently, a more systematic and transparent approach has been set out (5, 9–11). The NNR2022 project has developed an open and transparent process for selecting topics for *de novo* SRs, which builds on and further extends these procedures.

The process of selection of topics for SRs is important since these topics are selected in areas where it is possible or conceivable that the DRVs and FBDGs will be adjusted compared to the previous edition of NNR. Whilst this process never can be totally objective, the NNR2022 Committee has strived to select topics with the best intentions and based on the best of our knowledge, without ideological, commercial, political, or other types of subjective biases.

**Fig. 1:**
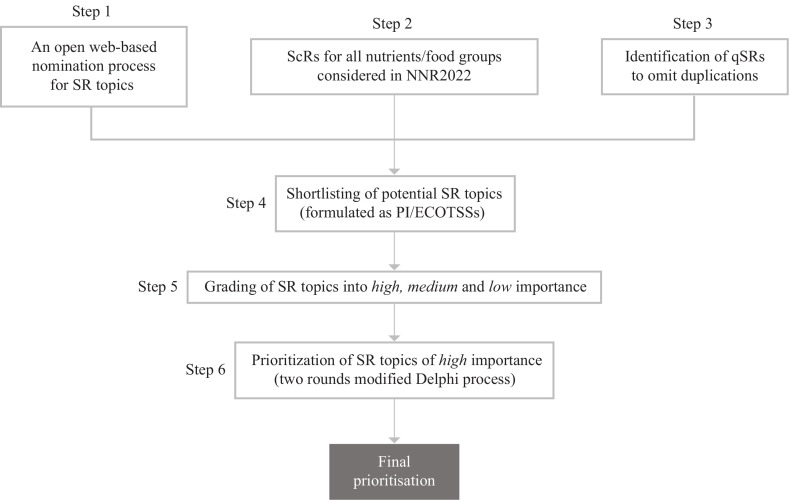
Multi-step process for prioritisation of topics for systematic reviews.

This paper describes the results of the six-step procedure to identify topics that will be prioritised for *de novo* SRs by the NNR2022 project (Fig. 1).

## Step 1. An open web-based nomination process for SR topics

An open nomination of topics amongst scientists, health professionals, national food and health authorities, food manufacturers, other stakeholders and the general population was organised. The nomination process was anonymous to reduce the risk of inherent bias by the NNR2022 Committee. For transparency, the results of the process are reported in this publication.

The open nomination process at the official NNR2022 website (12) was announced through press releases as well as emails to many hundred organisations, authorities, academic institutions, scientists and stakeholders in early September 2019. Deadline for the submission of topics was December 31, 2020. The submitted nominations consisted of three components: 1) a cover letter with a rationale and a description of why a review on a specific topic was warranted and how it related to health issues in Nordic and Baltic populations; 2) a list of references for scientific papers; and 3) a simple ‘PI/ECOTSS’ statement covering the elements ‘population’, ‘intervention/exposure’, ‘outcome’, ‘timing’, ‘setting’ and ‘study design’.

A total of 45 nominations with suggestion for more than 200 exposure–outcome pairs were received. Two nominations were excluded because they were incomplete; they were more like comments (see the complete list at the NNR2022 project website (12)). Forty-three of the nominations fulfilled all elements described earlier. The complete list of nominations, with their rationale and arguments, is available on the NNR2022 project website (12) and as an Electronic Supplementary Table 1. All submissions were considered by the NNR2022 Committee. Several of the nominations were overlapping, and some nominations needed to be interpreted and translated to a scientific question by the NNR2022 Committee. The NNR Committee developed a summary table of the nominations, where overlapping nominations were combined, that represents 43 exposure–outcome pairs (Table 1).

**Table 1:** Nomination of topics for systematic reviews from open call

Topic	Population	Intervention	Outcome	Timing
Obesity	Adults with body mass index (BMI) > 30	Avoidance obesogenic foods	Narrower waist, lower level of triglycerides	Lifetime
Plant-based, vegetarian and vegan diets	General population	Omega-3 fatty acids, eicosapentaenoic acid (EPA) and docosahexaenoic acid (DHA)	Heart health and cognitive function	Years
	General population (all age groups)	Plant-based diet and dietary supplements	Various health effects (obesity, diabetes, several cancers and heart disease) and vitamin deficiency	Short and long term
	Adults	Plant protein intake versus animal protein intake	Health effect (total mortality, diabetes type 2, all cancers and cardiovascular disease)	Weeks Randomized controlled trials (RCTs)and years (cohorts)
	Healthy children (including infants, babies and toddlers) in the Nordic countries	Vitamin B^12^ intake from foods (fortified foods) and supplements up to RDI	Vitamin B^12^ status, cognitive function (growth and development)	Years
	Children and women of childbearing age	Intake of plant-based foods	Iron status/iron absorption/iron bioavailability	Short term
	Healthy children and adults	Intake of foods containing plant protein isolates including soy protein isolates	Blood (plasma) concentrations of amino acids, lipids and glucose/insulin	Short term
	Children and pregnant and lactating women	Plant-based diet	All possible outcomes, but especially growth, neurological and cognitive developments	NA

Detection and correction of vitamin- and mineral deficiencies – biomarkers of intake	Adults	Assessment of vitamin and mineral status and need of supplementation	Restored adequate vitamin status	Months

Sustainability, and environmental and health impacts of foods and diets in the Nordic countries	General population	Potatoes	General health indicators and sustainability	Lifetime
	Nordic countries (including all age groups, gender and socio-economic groups)	Dietary patterns and specific food groups	Environmental impact (e.g. climate impact, eutrophication potential, acidification potential, land use demand, etc.) by using life cycle assessment (health outcomes not stated)	Not stated
	General and healthy populations in the Nordic countries	Nordic diet (foods primarily produced in the Nordics) whole food/whole sustainable diet approach	Nutrient intake (protein, vitamin D, calcium, riboflavin, vitamin B^12^, folate, iodine, selenium and zinc), long-term effects on public health and specific health parameters, biological diversity, animal welfare, responsible use of antibiotics in animal food production, carbon sequestration, responsible use of pesticides and use of land and water	>4 weeks

Inclusion of fruit-juice in FBDG	General population (distinguish in terms of BMI, age and gender)	Consumption of different volumes of pure fruit juice/compared to placebo/sugar sweetened fruit juice. May be consumed with a meal that induces inflammation	CRP and inflammatory cytokines	Short (hours) and long term (weeks)

Vitamin D requirements	Children and adolescents, fair and dark skinned in Nordic countries, including arctic areas	Intake of vitamin D	Vitamin D status	Long term
	Prepubertal children with fair and dark skin living in northern Europe	Vitamin D supplementation	Vitamin D status, calcium, PTH, cardiometabolic markers and BMI	>3 months
	Preschool children (1–5 years) with light versus dark skin colour	Requirement of vitamin D	Vitamin D status	Not stated

Meal pattern, timing and frequency, and regularity of meals/meal patterns	Children, adults and older adults	Meal pattern	Obesity related, unintentional weight loss/risk for malnutrition	Long term
	Children and adults	Timing/frequency/regularity of meals	Cardio metabolic health markers, body weight, obesity, lipid profile, insulin resistance and blood pressure	Not stated

Synbiotics in infant formula in treatment of cowmilk allergy	Infants consuming cowmilk formula	Intake of pre- and probiotics	Asthma, gastrointestinal disorders and eczema	Years

Degree of processing	General population	Reduction in intake of ultra-processed foods	Prevention of all diet-related Noncommunicable diseases (NCDs)	Long term
	All population groups	Intake of ultra-processed foods	Diet-related chronic diseases and diet quality	Lifetime

Diet in the elderly	Old adults (>75 years)	Weight change	Diabetes mellitus type 2, mortality and sarcopenic obesity?	Years
	Elderly population, aged 65 years or more	Energy, protein and B12	Risk of malnutrition, malnutrition, cost of malnutrition or its risk, morbidity, mortality and recovery	Years, lifetime

Vitamin K requirements (K1 and K2)	Healthy general population (all ages and different ethnicity)	Intake of vitamin K-rich foods or vitamin K supplement. Vitamin K1 and K2 should be examined separately. Comparators: diets low in total vitamin K/vitamin K1/vitamin K2, and supplements without these vitamins	Different health outcomes of vitamin K1 and K2, for example cardiovascular metabolism, bone health and diabetes	The timing varies
	Different populations, but primarily healthy humans, both genders, a broad range of age and ethnicity	Intervention: K2-rich foods or K2 supplement versus placebo, intervention diet versus subjects’ normal diets, lower versus upper percentiles	1) Vitamin K function with respect to its cofactor role in the carboxylation process of vitamin K-dependent proteins, amongst them matrix Gla protein (MGP), osteocalcin, and Gla-rich protein (GRP), and possible health effects. 2) Vitamin K function with respect to its cofactor role in muscle protein synthesis. 3) Vitamin K function with respect to its cofactor role in cardiovascular metabolism	A minimum of 4 weeks

Milk and dairy products and fat /dairy matrix	General population; different genders, ages, ethnicities, and health status	Intake of different dairy products in various amounts. Comparator(s): lower versus upper quartile	Cardiovascular disease and diabetes type 2 and their risk markers	Depends on study type
	Humans, both genders, different ranges of age, ethnicity and cardiovascular health status (not critically ill)	Intake of dairy food groups, different levels, for example: 1) full fat cheese versus low fat cheese, plus control group with no cheese intake; 2) full fat milk versus low fat milk, plus control group with no milk intake; 3) full fat yoghurt versus low fat yoghurt, plus control group with no yoghurt intake	LDL, ox LDL, VLDL, HDL, adiponectin. HbA1c and IL-6	Minimum 4 weeks
	The healthy population – all ages	Dairy fat	Adequate nutrient intake	Lifetime

Complementary feeding	0–2 years age, 3–5 years of age	Intake of different protein sources, sugar and sugary foods, water and other fluids, fruit and vegetables, fish and other sources of omega 3; amount of gluten at introduction and infancy, dose and timing of food allergens, meal order and snacking; effects of different parenting styles and responsive feeding	Overweight/obesity iron deficiency, neurodevelopment, vitamin D status, dental caries and allergies	Years

Choline	The Norwegian population, all ages	Intake of choline and all choline forms	Develop dietary recommendations	Years

Omega-3 fatty acid intake	Children, and pregnant and lactating women	Omega-3 fatty acids	All possible health outcomes, growth, neurological and cognitive developments and serum lipids	Lifetime

Intake of whole grains	General population, especially in the Nordic countries	Whole grain	Incident of coronary heart disease, stroke, type 2 diabetes, obesity, breast cancer, colorectal cancer, pancreatic cancer, gastric cancer, endometrial cancer, prostate cancer and mortality from all causes, respiratory diseases, infectious diseases and all non-cardiovascular and non-cancer causes	>5 years

Eggs and heart health	Adults (18 years of age or older)General population- Individuals with diabetes- Individuals with existing heart disease	Intervention: Eggs should be evaluated as a whole-food rather than examining constituents in eggs, such as cholesterol or choline. Comparators: another whole food (e.g. another protein source)	Cardiovascular disease (CVD) as a broad outcome classification coronary heart disease (CHD), coronary artery disease (CAD), ischemic heart disease. Cardiac events, including myocardial infarction. Cerebrovascular disease, including stroke. Both fatal and non-fatal outcomes should be considered	The analysis should be longitudinal in nature

Red and processed meat and cancer	Adults (18 years of age or older), who are free of chronic disease at baseline or study entry	Intervention: Red meat should be evaluated based on unprocessed and processed red meat items, and analyses that focus on this differentiation should be emphasised.Comparator: another whole food (e.g. another protein source) or to varying intake levels of red meat (e.g. daily intake vs. three times per week)	Total cancer incidence and mortality. Specific types of cancer, with an emphasis on colorectal cancer, which has been the most widely evaluated cancer type	The analysis should be longitudinal in nature

Gut microbiome	Infants in a birth cohort	Breast feeding	Composition of the gut microbiome, bodyweight, diabetes type 1 and celiac disease	5 and 10 years and maybe longer follow-up
	Adults and children	Plant-based diet	The growth of beneficial bacteria and the reduction of inflammation	For 3 months and 1 year
	Infants and children under 10 years of age	Intake of pro-, pre-, syn- and postbiotics	Gut microbiota, incidence and prevalence of non-communicable diseases	Years, lifetime
	Healthy adults	Different types of fibres	Composition of gut microbiome	Both short and long term (days/months)

Neurotoxic pesticide residues	Children (1–18 years)	Intake of common pesticides, including glyphosate and known neurotoxins	Mental health, learning disabilities, intellectual development, brain function, altered gut microbiota, anxiety, depression and child-learning capacity	Intervals from weeks to years

Chrono-biology and meal frequency	General population, adults and teenagers	Meal-time, meal frequency, temporal distribution and irregular meal patterns	Weight status, adiposity, diet quality and cardiovascular risk factors	Both short mechanistic studies and months/years

Vitamin- and mineral requirements during intravenous nutrition supply	Healthy adults	Use of intravenous nutrition (total parenteral nutrition)	Cover nutritional needs of macro- and micronutrients	Days to lifetime

Metabolic syndrome	Adults	Intake and distribution of macronutrients Intake of ultra-processed foods	Weight, metabolic syndrome and insulin resistance	Lifetime
		Intake of saturated fats	Cardiovascular disease and hard endpoints	Lifetime

The NNR2022 Committee formulated scientific questions based on the public call and the principles described in Arnesen et al. (ref 2–3).

Whilst only a limited number of topics made it through to the final list of SR prioritisations due to limited resources, all public nominations will be evaluated carefully by the NNR2022 Committee and various chapter experts when the DRVs and FBDGs are developed and formulated.

## Step 2. Scoping reviews on all nutrients and food groups considered in NNR2022

To develop candidate topics for prioritisation of *de novo* SRs, members of the NNR2022 Committee performed 51 scoping reviews (ScRs), one for each of the nutrients and food group chapters that will be part of the final NNR2022 report. An ScR is a relatively new approach to explore existing evidence (13). It differs from SRs both in its purpose and methodology. The purpose of an ScR is to provide an overview of available research without producing a synthesis and grading of total strength of evidence for a specific research question. An ScR should follow the procedures of the PRISMA Extension for Scoping Reviews (PRISMA-ScR) defined by the Equator Network (13). The methodology is much simpler than the extensive and more costly methodology for qualified SRs.

The objective of the 51 ScRs was to contribute to the shortlisting of topics. The major outcome of the ScRs was the formulation of shortlisted SR topics, formulated as PI/ECOTSS. Forty-nine topics were shortlisted based on the literature search. The literature search for the ScRs is presented in Electronic Supplementary Table 2.

When developing the search strategy for the ScRs, the aim was to identify possible topics that might be chosen for *de novo* SRs. We assumed that any topic with a significant amount of new data since the last edition of NNR would likely have been covered in a recent review article. We selected to set the bar at the level of ‘reviews’, rather than ‘systematic reviews’. By selecting reviews as the bar, we assume that we would pick up research activities that had not yet been dealt with in an SR. Thus, by choosing ‘reviews’, we have had a more open search with lower threshold than if we had selected ‘systematic reviews’.

In the NNR chapters, however, the initial ScR search string will be carefully adjusted and modified (e.g. by including ‘systematic reviews’, ‘meta-analysis’, ‘Mendelian randomisation studies’ and other types of relevant literature) when appropriate.

An evaluation of the results of the open public call (Electronic Supplementary Table 1) was included in each relevant ScR. Each ScR was considered by the NNR2022 Committee. The final version of the ScRs was formulated in a consensus process after several rounds of consultations in the NNR2022 Committee.

The criteria for shortlisting and prioritisation included evidence of significant new and relevant research since the previous edition of NNR (NNR2012) (14) and relevance to current public health concerns in the Nordic or Baltic countries (Box 1).

Box 1 Criteria for shortlisting and prioritisation of topics for de novo SRs.

- Relevance: The topic is within the scope of NNR2022.
- Within scope (examples): Healthy populations/individuals; prevention purposes (e.g. population health topics, clinically oriented topics are not included and people with increased genetic risk for chronic diseases are included); covers different age groups, and pregnant and lactating women; increased requirements during short-term mild infections; etc.
- Outside scope (examples): Long-term infections; malabsorption; various metabolic disturbances; treatment of persons with a sub-optimal nutritional status; clinical guidelines on dietary supplementation.
- Importance: The topic has new, relevant and significant data or is an emerging topic in an area of substantial public health interest or concern.
- Substantial public health concern (examples): Overweight, obesity and adiposity-related illness; metabolic syndrome and diabetes mellitus type 2; atherosclerotic cardiovascular diseases; cancer; osteoporosis; neurodegenerative diseases; mental health; oral health; multi-morbidity and mortality; reproductive health; optimal growth.
- Relevant and significant: Refers to the overall scientific quality of the evidence, the number of studies, consistency of results and whether new study results appear to expand the DRV- and FBDGs-related information available in the previous edition of NNR.
- Potential national impact: The SR may potentially inform national food and health policies and programs. An SR with the specific topic may result in a new or an adjustment of previous DRVs or FBDGs.
- If the research question is within the scope of NNR2022 and covers an outcome of substantial health concern to the Nordic and Baltic countries, then it has potential national impact. In other words, it may inform DRVs, FBDGs and national food and health policies and programs.
- No duplication: The topic is not currently addressed through other recent qualified SRs

## Step 3. Identification of qualified SRs to omit duplications

In order to omit duplication of recent qualified SRs, we established a process to identify relevant qualified SRs. The definition of a qualified SR was based on the inclusion and exclusion criteria (Box 2) pre-specified by the NNR2022 project (2–4).

Box 2 Inclusion and exclusion criteria for qSRs in the NNR2022 project.

**Inclusion criteria for SRs:**

- Commissioned by national food or health authorities, or international food and health organisation
- Authored by a group of multidisciplinary experts
- Consist of an original systematic review of the evidence for a nutrient/diet-health relationship
- Includes at least one nutrient/food topic and its relationship to at least one outcome related to a chronic disease or condition that is of public health interest in Nordic of Baltic countries; includes a clear description of the systematic review methodology, which should be similar to the methodology used NNR2022 (2, 3)
- Includes an assessment of the quality of primary studies
- Provides an evidence grade for the overall quality of the evidence
- English language
- Recent: Refer to SRs that have been published after the previous edition of the NNR

**Exclusion criteria for SRs:**

- Commissioned or sponsored by industry or an organisation with a business or ideological interest
- Authors with strong ties to industry or ideological organisations
- Later updated in another qualified SR on the same topic
- Focused on an outcome outside the scope of the NNR (e.g. disease management or food safety)

The search for qualified SRs was based on searches in PubMed/Medline and inspection of the websites of national and international food and health authorities as described by the Food and Agriculture Organization (FAO) of the United Nations (15). We also contacted the following major national food and health authorities and organisations directly for information on previous or planned SRs:

- National Academy of Sciences, Engineering and Medicine, USA
- Dietary Guidelines Advisory Committee, USA
- World Health Organization (WHO)
- World Cancer Research Fund (WCRF)
- European Food Safety Agency (EFSA)
- Scientific Advisory Committee on Nutrition (SACN), UK
- German Nutrition Society, Germany
- Health Council, The Netherlands
- National Health and Medical Research Council, Australia
- Ministry of Health, New Zealand
- Health Canada, Canada

All identified qualified SRs that fulfilled the inclusion and exclusion criteria are listed in Table 2.

**Table 2:** Qualified systematic reviews were identified based on the inclusion and exclusion criteria described in Box 1

Topic	Year	Authors/organisation (country)	Exposure(s)	Outcome(s)	Risk of bias assessment tool	SoE/evidence quality grading
1. Sodium and potassium intake	2018	Agency for Healthcare Research and Quality (AHRQ) (USA) (22)	Dietary sodium (sodium reduction) and potassium	Blood pressure, risk for cardiovascular diseases, all-cause mortality, renal disease and related risk factors, and adverse events	Cochrane RoB/Newcastle-Ottawa Scale (NOS). Some nutrition-specific items added (e.g. sodium intake assessment)	‘High’, ‘Moderate’, ‘Low’ or ‘Insufficient’. Based on: 1) Study limitations, 2) consistency, 3) directness, 4) precision and 5) reporting bias. Observational studies may be upgraded if very strong effects, a strong dose–response-relationship or if effects cannot be explained by uncontrolled confounding
2. Vitamin D and calcium	2014	AHRQ (USA) (23)	Vitamin D and/or calcium	Bone health, cardiovascular health, cancer, immune function, pregnancy, all-cause mortality and vitamin D status	CONSORT statement for RCTs, own checklist based on STROBE and nutrition-specific items	Grade A–B
3. Omega-3 fatty acids	2016	AHRQ (USA) (24)	Omega-3 fatty acids	Cardiovascular disease and risk factors	Cochrane RoB/NOS. Some nutrition-specific items added	‘High’, ‘Moderate’, ‘Low’ or ‘Insufficient’. Based on: 1) Study limitations, 2) consistency, 3) directness, 4) precision, 5) reporting bias and 6) number of studies
4. Omega-3 fatty acids	2016	AHRQ (USA) (25)	Omega-3 fatty acids	Maternal and child health: Gestational length, risk for preterm birth, birth weight, risk for low birth weight, risk for peripartum depression, risk for gestational hypertension/preeclampsia, postnatal growth, visual acuity, neurological development, cognitive development, autism spectrum disorder, ADHD, learning disorders, atopic dermatitis, allergies and respiratory disorders and adverse events	Cochrane RoB/NOS. Some nutrition-specific items added	‘High’, ‘Moderate’, ‘Low’ or ‘Insufficient’. Based on: 1) Study limitations, 2) consistency, 3) directness, 4) precision, 5) reporting bias and 6) number of studies
5. Nutrient reference values for sodium	2017	Australian Government Department of Health/New Zealand Ministry of Health (26)	Dietary sodium/sodium reduction	Blood pressure, cholesterol levels, stroke, myocardial infarction and total mortality	Cochrane RoB, modified	Grading of Recommendations Assessment, Development and Evaluation (GRADE) and National Health and Medical Research Council (NHMRC) level of evidence (from I to IV)
6. Dietary patterns	2020	Dietary Guidelines Advisory Committee (DGAC) (USA) (27)	Dietary patterns and macronutrient distribution	Growth, size, body composition, and/or risk of overweight or obesity	Cochrane RoB 2.0/Rob-Nobs*	Strength of evidence: ‘Strong’, ‘Moderate’, ‘Limited’ or ‘Not Assignable’; based on 1) risk of bias, 2) consistency, 3) directness, 4) precision and 5) generalisability
7. Dietary patterns (update of 2015 DGAC review)	2020	DGAC (USA) (28)	Dietary patterns	Cardiovascular disease, CVD risk factors (blood pressure, blood lipids)	Cochrane RoB 2.0/Rob-Nobs*	Strength of evidence: ‘Strong’, ‘Moderate’, ‘Limited’ or ‘Not Assignable’; based on 1) risk of bias, 2) consistency, 3) directness, 4) precision and 5) generalisability
8. Dietary patterns and risk of type 2 diabetes (update of 2015 DGAC review)	2020	DGAC (USA) (29)	Dietary patterns	Type 2 diabetes	Cochrane RoB 2.0/Rob-Nobs*	Strength of evidence: ‘Strong’, ‘Moderate’, ‘Limited’ or ‘Not Assignable’; based on 1) risk of bias, 2) consistency, 3) directness, 4) precision and 5) generalisability
9. Dietary patterns (update of 2015 DGAC review)	2020	DGAC (USA) (30)	Dietary patterns	Breast cancer, colorectal cancer, lung cancer and prostate cancer	Cochrane RoB 2.0/Rob-Nobs*	Strength of evidence: ‘Strong’, ‘Moderate’, ‘Limited’ or ‘Not Assignable’; based on 1) risk of bias, 2) consistency, 3) directness, 4) precision and 5) generalisability
10. Dietary patterns (update of 2015 DGAC review)	2020	DGAC (USA) (31)	Dietary patterns	Bone health, for example, risk of hip fracture and bone mineral density	Cochrane RoB 2.0/Rob-Nobs*	Strength of evidence: ‘Strong’, ‘Moderate’, ‘Limited’ or ‘Not Assignable’; based on 1) risk of bias, 2) consistency, 3) directness, 4) precision and 5) generalisability
11. Dietary patterns (update of 2015 DGAC review)	2020	DGAC (USA) (32)	Dietary patterns	Neurocognitive health, age-related cognitive impairment and dementia	Cochrane RoB 2.0/Rob-Nobs*	Strength of evidence: ‘Strong’, ‘Moderate’, ‘Limited’ or ‘Not Assignable’; based on 1) risk of bias, 2) consistency, 3) directness, 4) precision and 5) generalisability
12. Dietary patterns	2020	DGAC (USA) (33)	Dietary patterns	Sarcopenia	Cochrane RoB 2.0/Rob-Nobs*	Strength of evidence: ‘Strong’, ‘Moderate’, ‘Limited’ or ‘Not Assignable’; based on 1) risk of bias, 2) consistency, 3) directness, 4) precision and 5) generalisability
13. Dietary patterns	2020	DGAC (USA) (34)	Dietary patterns	Mortality	Cochrane RoB 2.0/Rob-Nobs*	Strength of evidence: ‘Strong’, ‘Moderate’, ‘Limited’ or ‘Not Assignable’; based on 1) risk of bias, 2) consistency, 3) directness, 4) precision and 5) generalisability
14. Dietary patterns during pregnancy	2020	DGAC (USA) (35)	Dietary patterns	Gestational weight gain	Cochrane RoB 2.0/Rob-Nobs*	Strength of evidence: ‘Strong’, ‘Moderate’, ‘Limited’ or ‘Not Assignable’; based on 1) risk of bias, 2) consistency, 3) directness, 4) precision and 5) generalisability
15. Dietary patterns during lactation	2020	DGAC (USA) (36)	Dietary patterns	Human milk composition and quantity	Cochrane RoB 2.0/Rob-Nobs*	Strength of evidence: ‘Strong’, ‘Moderate’, ‘Limited’ or ‘Not Assignable’; based on 1) risk of bias, 2) consistency, 3) directness, 4) precision and 5) generalisability
16. Folic acid from fortified foods and/or supplements during pregnancy and lactation	2020	DGAC (USA) (37)	Folic acid	Micronutrient status, gestational diabetes, hypertensive disorders during pregnancy, human milk composition and developmental milestones in child	Cochrane RoB 2.0/Rob-Nobs*	Strength of evidence: ‘Strong’, ‘Moderate’, ‘Limited’ or ‘Not Assignable’; based on 1) risk of bias, 2) consistency, 3) directness, 4) precision and 5) generalisability
17. Omega-3 fatty acids from supplements consumed before and during pregnancy and lactation	2020	DGAC (USA) (38)	Omega-3 from supplements	Risk of child food allergies and atopic allergic disease	Cochrane RoB 2.0/Rob-Nobs*	Strength of evidence: ‘Strong’, ‘Moderate’, ‘Limited’ or ‘Not Assignable’; based on 1) risk of bias, 2) consistency, 3) directness, 4) precision and 5) generalisability
18. Maternal diet during pregnancy and lactation	2020	DGAC (USA) (39)	Dietary patterns, food allergen (e.g. cow milk, eggs, fish, soybean, wheat, nuts, etc.)	Risk of child food allergies and atopic allergic diseases (e.g. atopic dermatitis, allergic rhinitis and asthma)	Cochrane RoB 2.0/Rob-Nobs*	Strength of evidence: ‘Strong’, ‘Moderate’, ‘Limited’ or ‘Not Assignable’; based on 1) risk of bias, 2) consistency, 3) directness, 4) precision and 5) generalisability
19. Exclusive human milk and/or infant formula consumption	2020	DGAC (USA) (40)	Human milk and/or infant formula	Overweight and obesity	Cochrane RoB 2.0/Rob-Nobs*	Strength of evidence: ‘Strong’, ‘Moderate’, ‘Limited’ or ‘Not Assignable’; based on 1) risk of bias, 2) consistency, 3) directness, 4) precision and 5) generalisability
20. Exclusive human milk and/or infant formula consumption	2020	DGAC (USA) (41)	Human milk and/or infant formula	Nutrient status (e.g. iron, zinc, iodine and vitamin B^12^ status)	Cochrane RoB 2.0/Rob-Nobs*	Strength of evidence: ‘Strong’, ‘Moderate’, ‘Limited’ or ‘Not Assignable’; based on 1) risk of bias, 2) consistency, 3) directness, 4) precision and 5) generalisability
21. Iron from supplements consumed during infancy and toddlerhood	2020	DGAC (USA) (42)	Iron from supplements	Growth, size and body composition	Cochrane RoB 2.0/Rob-Nobs*	Strength of evidence: ‘Strong’, ‘Moderate’, ‘Limited’ or ‘Not Assignable’; based on 1) risk of bias, 2) consistency, 3) directness, 4) precision and 5) generalisability
22. Vitamin D from supplements consumed during infancy and toddlerhood	2020	DGAC (USA) (43)	Vitamin D from supplements/fortified foods	Bone health (e.g. biomarkers, bone mass rickets and fracture) up to age 18 years	Cochrane RoB 2.0/Rob-Nobs*	Strength of evidence: ‘Strong’, ‘Moderate’, ‘Limited’ or ‘Not Assignable’; based on 1) risk of bias, 2) consistency, 3) directness, 4) precision and 5) generalisability
23. Beverage consumption	2020	DGAC (USA) (44)	Beverages (milk, juice, sugar-sweetened beverages, low and no-calorie beverages vs. water)	Growth, size, body composition and risk of overweight and obesity	Cochrane RoB 2.0/Rob-Nobs*	Strength of evidence: ‘Strong’, ‘Moderate’, ‘Limited’ or ‘Not Assignable’; based on 1) risk of bias, 2) consistency, 3) directness, 4) precision and 5) generalisability
24. Beverage consumption during pregnancy	2020	DGAC (USA) (45)	Beverages (milk, tea, coffee, sugar-sweetened/low- or no-calorie sweetened beverages and water)	Birth weight	Cochrane RoB 2.0/Rob-Nobs*	Strength of evidence: ‘Strong’, ‘Moderate’, ‘Limited’ or ‘Not Assignable’; based on 1) risk of bias, 2) consistency, 3) directness, 4) precision and 5) generalisability
25. Alcohol consumption	2020	DGAC (USA) (46)	Alcoholic beverages (type and drinking pattern)	Mortality	Cochrane RoB 2.0/Rob-Nobs*	Strength of evidence: ‘Strong’, ‘Moderate’, ‘Limited’ or ‘Not Assignable’; based on 1) risk of bias, 2) consistency, 3) directness, 4) precision and 5) generalisability
26. Added sugars (update of 2015 DGAC review)	2020	DGAC (USA) (47)	Added sugars; sugar-sweetened beverages	Cardiovascular disease, CVD mortality and CVD risk factors	Cochrane RoB 2.0/Rob-Nobs*	Strength of evidence: ‘Strong’, ‘Moderate’, ‘Limited’ or ‘Not Assignable’; based on 1) risk of bias, 2) consistency, 3) directness, 4) precision and 5) generalisability
27. Types of dietary fat	2020	DGAC (USA) (48)	Types of fatty acids, individual fatty acids (e.g. ALA, DHA), dietary cholesterol or food sources of types of fat (e.g. olive oil for MUFA, butter for SFA)	Cardiovascular disease outcomes and intermediate outcomes (blood lipids and blood pressure)	Cochrane RoB 2.0/Rob-Nobs*	Strength of evidence: ‘Strong’, ‘Moderate’, ‘Limited’ or ‘Not Assignable’; based on 1) risk of bias, 2) consistency, 3) directness, 4) precision and 5) generalisability
28. Seafood consumption during pregnancy and lactation	2020	DGAC (USA) (49)	Maternal seafood/fish intake (e.g. fish, salmon, tuna, trout, tilapia; shellfish: shrimp, crab and oysters)	Neurocognitive development (e.g. cognitive and language development; behavioural development; attention deficit disorder; autism spectrum disorder) in the child	Cochrane RoB 2.0/Rob-Nobs*	Strength of evidence: ‘Strong’, ‘Moderate’, ‘Limited’ or ‘Not Assignable’; based on 1) risk of bias, 2) consistency, 3) directness, 4) precision and 5) generalisability
29. Seafood consumption during childhood and adolescence (up to 18 years of age)	2020	DGAC (USA) (50)	Seafood (e.g. fish, salmon, tuna, trout and tilapia; shellfish: shrimp, crab and oysters)	Neurocognitive development (e.g. cognition, depression, dementia, psychomotor performance, behaviour disorders, autism spectrum disorder, mental health … academic achievement)	Cochrane RoB 2.0/Rob-Nobs*	Strength of evidence: ‘Strong’, ‘Moderate’, ‘Limited’ or ‘Not Assignable’; based on 1) risk of bias, 2) consistency, 3) directness, 4) precision and 5) generalisability
30. Seafood consumption during childhood and adolescence (up to 18 years of age)	2020	DGAC (USA) (51)	Seafood (e.g. salmon, tuna, trout and tilapia; shellfish: shrimp, crab and oysters)	Cardiovascular disease (and blood lipids or blood pressure)	Cochrane RoB 2.0/Rob-Nobs*	Strength of evidence: ‘Strong’, ‘Moderate’, ‘Limited’ or ‘Not Assignable’; based on 1) risk of bias, 2) consistency, 3) directness, 4) precision and 5) generalisability
31. Frequency of eating	2020	DGAC (USA) (52)	Eating frequency	Overweight and obesity	Cochrane RoB 2.0/Rob-Nobs*	Strength of evidence: ‘Strong’, ‘Moderate’, ‘Limited’ or ‘Not Assignable’; based on 1) risk of bias, 2) consistency, 3) directness, 4) precision and 5) generalisability
32. Frequency of eating	2020	DGAC (USA) (53)	Eating frequency	Cardiovascular disease	Cochrane RoB 2.0/Rob-Nobs*	Strength of evidence: ‘Strong’, ‘Moderate’, ‘Limited’ or ‘Not Assignable’; based on 1) risk of bias, 2) consistency, 3) directness, 4) precision and 5) generalisability
33. Frequency of eating	2020	DGAC (USA) (54)	Eating frequency	Type 2 diabetes	Cochrane RoB 2.0/Rob-Nobs*	Strength of evidence: ‘Strong’, ‘Moderate’, ‘Limited’ or ‘Not Assignable’; based on 1) risk of bias, 2) consistency, 3) directness, 4) precision and 5) generalisability
34. Dietary patterns and long-term food sustainability and related food security	2015	DGAC (USA) (55)	Dietary patterns	Environmental impact	NEL Bias assessment tool	‘Strong’, ‘Moderate’, ‘Limited’, ‘Expert opinion only’, ‘Not assignable’; based on 1) risk of bias, 2) consistency, 3) quantity, 4) impact and 5) generalisability
35. Sodium intake in children	2015	DGAC (USA) (55)	Dietary sodium	Blood pressure	NEL Bias assessment tool	‘Strong’, ‘Moderate’, ‘Limited’, ‘Expert opinion only’, ‘Not assignable’; based on 1) risk of bias, 2) consistency, 3) quantity, 4) impact and 5) generalisability
36. Sodium intake	2015	DGAC (USA) (55)	Dietary sodium	Cardiovascular disease	NEL Bias assessment tool	‘Strong’, ‘Moderate’, ‘Limited’, ‘Expert opinion only’, ‘Not assignable’; based on 1) risk of bias, 2) consistency, 3) quantity, 4) impact and 5) generalisability
37. Added sugars	2015	DGAC (USA) (55)	Added sugars and sugar-sweetened beverages	CVD, CVD mortality, hypertension, blood pressure, cholesterol and triglycerides	NEL Bias assessment tool	‘Strong’, ‘Moderate’, ‘Limited’, ‘Expert opinion only’, ‘Not assignable’; based on 1) risk of bias, 2) consistency, 3) quantity, 4) impact and 5) generalisability
38. Carbohydrates	2012	German Nutrition Society (DGE) (Germany) (56)	Total carbohydrates, sugars, sugar-sweetened beverages, dietary fibre, whole-grain and glycaemic index/load	Obesity, type 2 diabetes, dyslipidaemia, hypertension, metabolic syndrome, coronary heart disease and cancer	WHO level of evidence (Ia-Ic, IIa-IIb) based on study design	WHO/WCRF (convincing, probable, possible and insufficient) /(convincing, probable, limited-suggestive, limited - no conclusion)
39. Fatty acids	2015	DGE (Germany) (57)	Dietary fats	Adiposity, type 2 diabetes, dyslipidaemia/hyperlipidaemia, blood pressure, cardiovascular diseases, metabolic syndrome and cancer	WHO level of evidence (Ia-Ic, IIa-IIb) based on study design	WHO/WCRF (convincing, probable, possible and insufficient) /(convincing, probable, limited-suggestive, limited - no conclusion)
40. Dietary reference values for sodium	2019	EFSA (58)	Sodium intake, as 24 h sodium excretion (i.e. not self-reported)	Blood pressure, CVD, bone mineral density, osteoporotic fractures and sodium balance	OHAT/NTP risk of bias tool (based on AHRQ, Cochrane, Clarity, etc.): selection, performance, attrition, detection and selective reporting bias	‘Uncertainty analysis’ based on consistency, precision, internal and external validities, etc.
41. Dietary references values for copper	2012	EFSA, review by ANSES (France) (59)	Copper	Copper status, bioavailability, cardiac arrythmia, cancer, arthritis, cognitive function, respiratory disease and cardiovascular mortality	EURRECA system (high, moderate, low or unclear), partly based on Cochrane	Consistency, strength and quality of the studies (see Dhonukshe-Rutten et al. 2013 (60) and EFSA, 2010 (principles) (61))
42. Dietary reference values for riboflavin	2014	EFSA, review by Pallas Health Research (Netherlands) (62)	Riboflavin	Riboflavin status, biomarkers, cancer, mortality, bone health, infant health, etc.	EURRECA system (high, moderate, low or unclear), partly based on Cochrane	Consistency, strength and quality of the studies (see Dhonukshe-Rutten et al. 2013 (60) and EFSA, 2010 (principles) (61))
43. Dietary reference values for phosphorus, sodium and chloride	2013	EFSA, review by Pallas Health Research (Netherlands) (63)	Phosphorus, sodium and chloride	Status, adequacy, health outcomes including cancer, CVD, kidney disease, all-cause and CVD mortality	EURRECA system (high, moderate, low or unclear), partly based on Cochrane	Consistency, strength and quality of the studies (see Dhonukshe-Rutten et al. 2013 (60) and EFSA, 2010 (principles) (61))
44. Dietary reference values for niacin, biotin and vitamin B6	2012	EFSA, review by Pallas Health Research (Netherlands) (64)	Niacin	Niacin/biotin/vitamin B^6^ status, adequacy, bioavailability, cancer, CVD, cognitive decline, infant health, all-cause mortality, etc.	EURRECA system (high, moderate, low or unclear), partly based on Cochrane	Consistency, strength and quality of the studies (see Dhonukshe-Rutten et al. 2013 (60) and EFSA, 2010 (principles) (61))
45. Milk and dairy consumption during pregnancy	2012	NNR: Brantsæter et al. (65)	Milk and dairy products	Birth weight, foetal growth, large for gestational age and small for gestational age	NNR quality assessment tool (rated A, B or C)	WCRF (convincing, probable, limited – suggestive, limited – no conclusion)
46. Dietary	2013	NNR: Dommelof et al. (66)	Iron intake at different life stages	Requirements for adequate growth, development and maintenance of health (anaemia, cognitive/behavioural function, cancer and cardiovascular disease)	NNR quality assessment tool (rated A, B or C)	WCRF (convincing, probable, limited – suggestive, limited – no conclusion)
47. Dietary macronutrients	2012	NNR: Fogelholm et al. (67)	Dietary macronutrient consumption	Primary prevention of long-term weight/WC/body fat changes, or changes after weight loss	NNR quality assessment tool (rated A, B or C)	WCRF (convincing, probable, limited – suggestive, limited – no conclusion)
48. Iodine	2012	NNR: Gunnarsdottir et al. (68)	Iodine status	Requirements for adequate growth, development and maintenance of health (pregnancy, childhood development, thyroid function and metabolism)	NNR quality assessment tool (rated A, B or C)	WCRF (convincing, probable, limited – suggestive, limited – no conclusion)
49. Protein intake from 0 to 18 years of age	2013	NNR: Hörnell et al. (69)	Protein intake in infancy and childhood	Functional/clinical outcomes and risk factors (including serum lipids, glucose and insulin, blood pressure, body weight and bone health)	NNR quality assessment tool (rated A, B or C)	WCRF (convincing, probable, limited – suggestive, limited – no conclusion)
50. Breastfeeding, introduction of other foods and effects on health	2013	NNR: Hörnell et al. (70)	Breastfeeding and introduction of other foods	Growth in infancy, overweight and obesity, atopic disease, asthma, allergy, health and disease outcomes, including infectious disease, cognitive and neurological developments, CVD, cancer, diabetes, blood pressure, glucose tolerance and insulin resistance)	NNR quality assessment tool (rated A, B or C)	WCRF (convincing, probable, limited – suggestive, limited – no conclusion)
51. Vitamin D	2013	NNR: Lamberg-Allardt et al. (71)	Vitamin D	Dietary reference values, vitamin D status, requirements for adequate growth, development and maintenance of health, upper limits, pregnancy outcomes, bone health, cancer, diabetes, obesity, total mortality, CVD and infections	NNR quality assessment tool (rated A, B or C)	WCRF (convincing, probable, limited – suggestive, limited – no conclusion)
52. Protein intake in elderly populations	2014	NNR: Pedersen et al. (72)	Protein intake in elderly populations	Dietary requirements (nitrogen balance), muscle mass, bone health, physical training and potential risks	NNR quality assessment tool (rated A, B or C)	WCRF (convincing, probable, limited – suggestive, limited – no conclusion)
53. Protein intake in adults	2013	NNR: Pedersen et al. (73)	Protein intake, protein sources	Dietary requirements, markers of functional or clinical outcomes (including serum lipids, glucose and insulin and blood pressure), pregnancy or birth outcomes, CVD, body weight, cancer, diabetes, fractures, renal function, physical training, muscular strength and mortality	NNR quality assessment tool (rated A, B or C)	WCRF (convincing, probable, limited – suggestive, limited – no conclusion)
54. Dietary fat	2014	NNR: Schwab et al. (74)	Types of dietary fat	Body weight, diabetes, CVD, cancer, all-cause mortality and risk factors (including serum lipids, glucose and insulin, blood pressure and inflammation)	NNR quality assessment tool (rated A, B or C)	WCRF (convincing, probable, limited – suggestive, limited – no conclusion)
55. Sugar consumption	2012	NNR: Sonestedt et al. (75)	Sugar intake and sugar-sweetened beverages	Type 2 diabetes, CVD, metabolic risk factors (including glucose tolerance, insulin sensitivity, dyslipidaemia, blood pressure, uric acid and inflammation) and all-cause mortality	NNR quality assessment tool (rated A, B or C)	WCRF (convincing, probable, limited – suggestive, limited – no conclusion)
56. Calcium	2013	NNR: Uusi-Rasi et al. (76)	Calcium	Calcium requirements, upper intake level, adequate growth, development and maintenance of health, bone health, muscle strength, cancer, autoimmune diseases, diabetes, obesity/weight control, all-cause mortality and CVD	NNR quality assessment tool (rated A, B or C)	WCRF (convincing, probable, limited – suggestive, limited – no conclusion)
57. Health effects associated with foods characteristic of the nordic diet	2013	NNR: Åkesson et al. (77)	Potatoes, berries, whole grains, dairy products and red meat/processed meat	CVD incidence and mortality, Type 2 diabetes, inflammatory factors, colorectal, prostate and breast cancers, bone health and iron status	NNR quality assessment tool (rated A, B or C)	WCRF (convincing, probable, limited – suggestive, limited – no conclusion)
58. Carbohydrates	2015	SACN (UK) (78)	Total carbohydrates, sugars, sugar-sweetened food/beverages, starch, starchy foods, dietary fibre and glycemic index/load	Obesity, cardio-metabolic health, energy intake, colorectal health (cancer, IBS, constipation) and oral health	Cochrane RoB and observational studies: no formal grading, but markers of study quality = cohort size, attrition, follow-up time, sampling method and response rate, participant characteristics and dietary intake assessment	‘Adequate’, ‘moderate’, ‘limited’ (own grading system based on study quality, study size, methodological considerations and specific criteria to upgrade, for example, dose-response relationship)
59. Alcohol	2018	WCRF (79)	Alcoholic drinks (beer, wine, spirits, fermented milk, mead and cider)	Cancer (including of mouth, pharynx and larynx, oesophagus, liver, colorectal, breast, kidney, stomach, lung, pancreas and skin)	Cochrane RoB/NOS	WCRF (convincing, probable, limited-suggestive, limited - no conclusion)
60. Body fatness and weight gain	2018	WCRF (80)	Body fatness: BMI, waist circumference, W-H ratio, adult weight gain	Cancer (including of mouth, pharynx and larynx, oesophagus, liver, colorectal, breast, kidney, stomach, lung, pancreas, gallbladder, ovary, prostate, etc.)	Cochrane RoB/NOS	WCRF (convincing, probable, limited-suggestive, limited - no conclusion)
61. Energy balance	2018	WCRF (81)	Dietary patterns, foods, macronutrients, energy density, lactation and physical activity	Weight gain, overweight and obesity	From NICE (2014) report (low, moderate and high quality) (ref. obesity: identification, assessment and management of overweight and obesity in)	WCRF
62.Height and birthweight	2018	WCRF (82)	Attained height, growth and birthweight	Cancer (including of mouth, pharynx and larynx, oesophagus, liver, colorectal, breast, kidney, stomach, lung, pancreas, gallbladder, ovary, prostate, etc.)	Cochrane RoB/NOS	WCRF
63. Lactation	2018	WCRF (83)	Lactation	Cancer (including of breast, ovary, etc.) in the mother who is breastfeeding	Cochrane RoB/NOS	WCRF
64. Meat, fish and dairy	2018	WCRF (84)	Meat, fish and dairy products; haem iron; diets high in calcium	Cancer (including of mouth, pharynx and larynx, oesophagus, liver, colorectal, breast, kidney, stomach, lung, pancreas, gallbladder, ovary, prostate, etc.)	Cochrane RoB/NOS	WCRF
65. Non-alcoholic drinks	2018	WCRF (85)	Non-alcoholic drinks: water/arsenic in drinking water, coffee, tea and mate	Cancer (including of mouth, pharynx and larynx, oesophagus, liver, colorectal, breast, kidney, stomach, lung, pancreas, gallbladder, ovary, prostate, etc.)	Cochrane RoB/NOS	WCRF
66. Other	2018	WCRF (86)	Dietary patterns, macronutrients, micronutrients in foods or supplements, glycaemic load	Cancer (including of mouth, pharynx and larynx, oesophagus, liver, colorectal, breast, kidney, stomach, lung, pancreas, gallbladder, ovary, prostate, etc.)	Cochrane RoB/NOS	WCRF
67. Physical activity	2018	WCRF (87)	Physical activity, types of physical activity and intensity	Cancer (including of mouth, pharynx and larynx, oesophagus, liver, colorectal, breast, kidney, stomach, lung, pancreas, gallbladder, ovary, prostate, etc.)	Cochrane RoB/NOS	WCRF
68. Preservation and processing	2018	WCRF (88)	Salting, curing, fermentation, smoking; processed meat and fish	Cancer (including of mouth, pharynx and larynx, oesophagus, liver, colorectal, breast, kidney, stomach, lung, pancreas, gallbladder, ovary, prostate, etc.)	Cochrane RoB/NOS	WCRF
69. Wholegrains, fruit and vegetables	2018	WCRF (89)	Wholegrains, pulses (legumes), vegetables, fruits, dietary fibre, aflatoxins, beta-carotene, carotenoids, vitamin C and isoflavones	Cancer (including of mouth, pharynx and larynx, oesophagus, liver, colorectal, breast, kidney, stomach, lung, pancreas, gallbladder, ovary, prostate, etc.)	Cochrane RoB/NOS	WCRF
70. Sugars	2015	WHO (90)	Total, added or free sugars, sugar-sweetened beverages, fruit juice	Body weight, body fatness and dental caries	Cochrane RoB/cohort studies: own	GRADE
71. Sodium	2012	WHO (91)	Sodium intake/reduced sodium intake and sodium excretion	Cardiovascular diseases, all-cause mortality, blood pressure, renal function, blood lipids and potential adverse effects	Cochrane RoB	GRADE
72. Potassium	2012	WHO (Aburto et al. 2013) (92)	Potassium intake, 24 h urinary potassium excretion	Blood pressure, cardiovascular diseases, all-cause mortality, cholesterol, noradrenaline, creatinine and side effects	Cochrane RoB	GRADE
73. Trans-fats	2016	WHO (de Souza et al. 2015 (93); Brouwer et al. 2016) (94)	Trans fatty acids	All-cause mortality, cardiovascular disease, type 2 diabetes and blood lipids	Cochrane RoB (for TFA and blood lipids)/NOS	GRADE
74. Saturated fats	2016	WHO (Hooper, 2015; Mensink, 2016; Te Morenga 2017) (95–97)	Saturated fat reduction	Cardiovascular disease, mortality, blood lipids, other risk factors and growth (children)	Cochrane RoB, other potential sources of bias, for example, compliance	GRADE
75. Carbohydrate quality	2019	WHO (Reynolds et al., Lancet) (98)	Markers of carbohydrate quality, that is, dietary fibre, glycaemic index/load and whole grains	All-cause mortality, coronary heart disease, stroke, type 2 diabetes, colorectal cancer, adiposity-related cancers, adiposity, fasting glucose/insulin/insulin sensitivity/HbA1c, blood lipids and blood pressure	Cochrane RoB/NOS/ROBIS	GRADE
76. Omega-3, omeg-6 and polyunsaturated fat	2020	Brainard et al. (99)	Higher versus lower omega-3, omega-6 or polyunsaturated fats	New neurocognitive illness, newly impaired cognition and/or continuous measures of cognition	Cochrane RoB	GRADE

## Step 4: Formulation and shortlisting of PI/ECOTSS statements

All shortlisted topics from the ScRs and the public call were formulated by the NNR2022 Committee as initial PI/ECOTSS statements (2–4). The shortlisted PI/ECOTSS statements were then compared with topics covered in the list of qSRs (Table 2), and overlapping PI/ECOTSS statements, which had not been removed in a previous stage, were excluded from the shortlisting. The initial formulation of PI/ECOTSS statements was adjusted by the NNR2022 Committee during several steps of this process to improve the precision of the scientific question. Consultation with topic experts, the members of the NNR SR Centre and the Scientific Advisory Group was helpful in formulating the final PI/ECOTSS statements. Elimination of PI/ECOTSS statements due to overlap with qSRs was continuously updated in accordance with the ongoing adjustments in PI/ECOTSS statements.

Table 3 presents the 52 PI/ECOTSS statements that were shortlisted.

**Table 3:** Shortlisted topics for systematic reviews

Topic								
Iron								
Population	Intervention or exposure	Comparators	Outcomes	Timing	Setting	Study design	Ranking	Argument for ranking
Adults+40 years	Iron intake and status Several biomarkers of status available for example serum ferritin	Low versus high intake Different levels of iron status, for example, deficiency or excess	Type 2 diabetes and markers of glucose metabolism	Minimum 12 months follow-up in cohort studies. Minimum 4-week intervention in intervention studies	Relevant for the general population in the Nordic and Baltic countries	Prospective cohort studies Intervention studies randomized controlled trials (RCTs)	Low	Public health concern. New evidence unlikely to influence DRV
Pregnant women	Iron intake and status Several biomarkers of status available for example serum ferritin	Low versus high intake Different levels of iron status, for example, deficiency or excess	Gestational diabetes	Minimum 12 months follow-up in cohort studies. Minimum 4-week intervention in intervention studies	Relevant for the general population in the Nordic and Baltic countries	Cohort studies Intervention studies	Low	New evidence unlikely to influence DRV
Children First years of life	Iron intake and status Several biomarkers of status available for example serum ferritin	Low versus high intake Different levels of iron status, for example, deficiency or excess	Mental and psychomotor development	Minimum 12 months follow-up in cohort studies. Minimum 4-week intervention in intervention studies	Relevant for the general population in the Nordic and Baltic countries	Cohort studies Intervention studies	Low	New evidence unlikely to influence DRV

Magnesium								

Population	Intervention or exposure	Comparators	Outcomes	Timing	Setting	Study design	Ranking	Argument for ranking

Adults	Mg intake/status	Low versus high, dose response to find protective level	Risk of type 2 diabetes and markers of glucose metabolism	Minimum 12 months follow-up in cohort studies. Minimum 4-week intervention in intervention studies	Relevant for the general population in the Nordic and Baltic countries	Prospective cohort studies Intervention studies	Low	The topic has new, relevant data in an area of substantial public health concern, but no good biomarkers of status. New evidence unlikely to influence DRV
Adults	Mg intake/status	Low versus high dose response to find protective level	Risk of CVD and indicators of CVD	Minimum 12 months follow-up in cohort studies. Minimum 4-week intervention in intervention studies	Relevant for the general population in the Nordic and Baltic countries	Prospective cohort studies Intervention studies	Low	The topic has new, relevant data in an area of substantial public health concern, but no good biomarkers of status. New evidence unlikely to influence DRV

Protein								

Population	Intervention or exposure	Comparators	Outcomes	Timing	Setting	Study design	Ranking	Argument for ranking

Adults	Plant protein intake	Animal protein intake	CVD and diabetes in prospective studies. CVD qualified surrogate endpoints and diabetes/insulin resistance/sensitivity in RCTs	Minimum 12 months for prospective studies and 1 month for RCTs, depending on outcome	Relevant for the general population in the Nordic and Baltic countries	RCT and prospective cohorts	High	The topic has new, relevant data in an area of substantial public health concern
Adults	Plant protein intake	Animal protein intake, different sources	Bone health (to be defined)	Five years for prospective studies and 1 month for RCTs	Relevant for the general population in the Nordic and Baltic countries	RCT and prospective cohorts	Low	The effect of type of protein was not considered a major driver of this public health issue
Older adults	Protein intake	Other macronutrients	Body composition and muscle strength	Minimum 12 months follow-up in cohort studies. Minimum 4-week intervention in intervention studies	Relevant for the general population in the Nordic and Baltic countries	RCTs and prospective cohorts	Medium	Total protein intake relevant issue for this age group, sources of protein, much less data. New guidelines, for example, ESPEN, suggest little new data to set recommendations
Children	1. Total protein intake 2. Amount and different sources of protein, for example, plant versus animal protein intake, dairy protein intake	Highest versus lowest protein intakes as defined by, for example, quartiles or risk difference per gram protein from one source relative to other sources	Anthropometry (length in cm and SDS, weight in kg and %), risk of overweight or obesity	Minimum 6 months follow-up in cohort studies. Minimum 4-week intervention in intervention studies (depending on the age of the child)	Relevant for Nordic setting (excludes, for example, populations with high prevalence of childhood malnutrition)	RCT and prospective cohorts	High	The topic has new, relevant data in an area of substantial public health concern
Adults	Protein isolates (dependent on a new search to confirm)	Wholefoods protein	Plasma concentrations of amino acids, lipids, glucose and insulin	Minimum 4-week intervention in intervention studies	Relevant for the general population in the Nordic and Baltic countries	RCT	High	The topic has new, relevant data in an area of substantial public health concern

Zinc								

Population	Intervention or exposure	Comparators	Outcomes	Timing	Setting	Study design	Ranking	Argument for ranking

Adults +40 years	Zinc intake and status	Low versus high dietary intake of zinc If available, status may be measured as plasma zinc concentration	Type 2 diabetes and markers of diabetes	Minimum 12 months follow-up in cohort studies. Minimum 4-week intervention in intervention studies	Relevant for the general population in the Nordic and Baltic countries	Prospective cohort studies and intervention studies	Medium	Despite public health importance of T2D, the limited evidence available suggests no association between zinc status and T2DM risk Supplemental zinc for the prevention of diabetes has been reviewed in a Cochrane SR
Adults +40 years	Zinc intake and status	Low versus high dietary intake of zinc If available, status may be measured as plasma zinc concentration	Cardiovascular disease	Minimum 12 months follow-up in cohort studies. Minimum 4-week intervention in intervention studies	Relevant for the general population in the Nordic and Baltic countries	Prospective cohort studies and intervention studies	Medium	Public health importance of CVD. Zinc has anti-oxidative stress and anti-inflammatory functions. Evidence of association
Adults +40 years	Zinc intake and status	Low versus high dietary intake of zinc If available, status may be measured as plasma zinc concentration	Digestive tract cancer	Minimum 12 months follow-up in cohort studies. Minimum 4-week intervention in intervention studies	Relevant for the general population in the Nordic and Baltic countries	Prospective cohort studies and intervention studies	Low	Zinc is not one of the exposures mentioned in the WCRF 3rd expert report as a risk factor for cancer. New evidence unlikely to influence DRV
Children first years of life	Zinc intake and status	Low versus high dietary intake of zinc If available, status may be measured as plasma zinc concentration	Growth and cognition	Minimum 12 months follow-up in cohort studies. Minimum 4-week intervention in intervention studies	Relevant for the general population in the Nordic and Baltic countries	Cohort studies Intervention studies	Low	WHO is planning an SRs on zinc for children aged 0–36 months

Dietary fibre								

Population	Intervention or exposure	Comparators	Outcomes	Timing	Setting	Study design	Ranking	Argument for ranking

Children	DF and sub-groups, for example, soluble and in-soluble. Or subgroups related to the fractions in chemical analyses Or depending on origin gain, pulses and vegetables fruits	High-low Dose-response	Bowel function* Energy availability Nutrient availability All including risks of high intake *Specific outcomes have to be identified	Short time/few days of follow-up, depending on study design and outcome	Relevant for the general population in the Nordic and Baltic countries	Prospective cohort studies, interventions and RCTs	High	Dietary fibre intake will increase with adherence to a more plant based and environmentally sustainable diet. The effect on children must be considered

Vegetables, fruits and berries								

Population	Intervention or exposure	Comparators	Outcomes	Timing	Setting	Study design	Ranking	Argument for ranking

Adults	F&V	No/low consumption and dose-response	T2D and CVD	Minimum 12 months for prospective studies and 1 month for RCTs, depending on outcome	Relevant for the general population in the Nordic and Baltic countries	Prospective cohort studies and interventions	High	More data since 2012 with potential to influence the quantitative recommendation
Adults	Sub-groups of vegs: dark green leafy and berries	No/low consumption and dose-response	T2D, CVD and bone health	Minimum12 months for prospective studies and 1 month for RCTs, depending on outcome	Relevant for the general population in the Nordic and Baltic countries	Prospective cohort studies and interventions	High	Intake will increase with adherence to a more plan-based and environmentally sustainable diet. Health effects must be considered
Adults	F&V	No/low consumption of	Wheezing and asthma	Minimum 12 months for prospective studies and 1 month for RCTs, depending on outcome	Relevant for the general population in the Nordic and Baltic countries	Prospective cohort studies and interventions	Low	New evidence unlikely to influence DRV
Adults	Potatoes	No/low consumption and dose-response	All-cause mortality, CVD, CHD, stroke, T2D, obesity and hypertension	Minimum 12 months for prospective studies and 1 month for RCTs, depending on outcome	General population	Prospective cohort studies and interventions	Low	Due to limited data. New evidence unlikely to influence DRV

Pulses and legumes								

Population	Intervention or exposure	Comparators	Outcomes	Timing	Setting	Study design	Ranking	Argument for ranking

Adults (≥18 years)	Pulses/legumes (subgroups if possible), exclude peanuts	No/low versus high consumption Dose-response	CVD and type 2 diabetes in prospective studies. CVD qualified surrogate endpoints and diabetes/insulin resistance/sensitivity in RCTs	Minimum 12 months for prospective studies and 1 month for RCTs, depending on outcome	Relevant for the general population in the Nordic and Baltic countries	Prospective cohort studies and interventions	High	High priority due to focus on sustainability of diets and not covered by NNR2012 Increasing consumption, greater variety and new studies Important to appraise this association since these foods are important as substitutes for meat
Adults	Pulses/legumes	No/low consumption of pulses and sub-groups Dose-response	Overweight	Minimum 12 months for prospective studies and 1 month for RCTs, depending on outcome	Relevant for the general population in the Nordic and Baltic countries	Prospective cohort studies and interventions	Low	New evidence unlikely to influence DRV. More studies may be needed
Adults	Soy/fermented soy products	No/low consumption soy/fermented soy products	Alzheimer’s disease/dementia/reproductive health/osteoporosis	Minimum 12 months for prospective studies and 1 month for RCTs, depending on outcome	Relevant for the general population in the Nordic and Baltic countries	Prospective cohort studies and interventions	Low	New evidence unlikely to influence DRV. More studies may be needed

Vitamin D								

Population	Intervention or exposure	Comparators	Outcomes	Timing	Setting	Study design	Ranking	Argument for ranking

Elderly 70+ years	Vitamin D	Placebo	Mortality	Minimum 12 months follow-up in cohort studies. Minimum 4-week intervention in intervention studies	Relevant for the general population in the Nordic and Baltic countries	RCTs, cohort studies and case–control studies	Low	New SRs are published, and mortality was included in NNR2012. New evidence unlikely to influence DRV
Adults 18–50 years	Vitamin D	Placebo	Cognition	Minimum 12 months follow-up in cohort studies. Minimum 4-week intervention in intervention studies	Relevant for the general population in the Nordic and Baltic countries	RCTs, cohort studies and case–control studies	Low	New SRs are published, but intervention studies are missing. The DO-HEALTH study, however, has included cognition as an outcome. New evidence unlikely to influence DRV

Vitamin D								

Population	Intervention or exposure	Comparators	Outcomes	Timing	Setting	Study design	Ranking	Argument for ranking

Elderly, adults, 50+ years	Vitamin D	Placebo	Musculo-skeletal health	Minimum 12 months follow-up in cohort studies. Minimum 4-week intervention in intervention studies	Relevant for the general population in the Nordic and Baltic countries	RCTs, cohort studies and case–control studies	Low	New SRs are published, but bone health/falls/muscle strength and included in NNR2012
Children, adults, 2–18 years	Vitamin D	Placebo	Respiratory infections	Minimum 12 months follow-up in cohort studies. Minimum 4-week intervention in intervention studies	Relevant for the general population in the Nordic and Baltic countries	RCTs, cohort studies and case–control studies	High	New SRs are published, and respiratory infections were not included in NNR2012
Women, 18–45 years	Vitamin D	Placebo	Pregnancy outcomes	Minimum 12 months follow-up in cohort studies. Minimum 4-week intervention in intervention studies	Pregnant and lactating women	RCTs, cohort studies and case–control studies	Low	New SRs are published, and pregnancy outcomes were included in NNR2012
Adults, 18–70+	Vitamin D	Placebo	Diabetes/metabolic syndrome	Minimum 12 months follow-up in cohort studies. Minimum 4-week intervention in intervention studies	Relevant for the general population in the Nordic and Baltic countries	RCTs, cohort studies and case–control studies	Low	New SRs are published, and diabetes was included in NNR2012
Children, adults and elderly, 2–70+	Vitamin D	Different doses	Dose-response relations	Minimum 12 months follow-up in cohort studies. Minimum 4-week intervention in intervention studies	Relevant for the general population in the Nordic and Baltic countries	RCTs, cohort studies and case–control studies	High	New SRs are published, and the dose-response relation is fundamental for all outcomes
Adults, 18–70+	Vitamin D	Polymorphism	Vitamin D status	Minimum 12 months follow-up in cohort studies. Minimum 4-week intervention in intervention studies	Relevant for the general population in the Nordic and Baltic countries		High	New SR are published, and genotypes were not included in NNR2012

Vitamin D								

Population	Intervention or exposure	Comparators	Outcomes	Timing	Setting	Study design	Ranking	Argument for ranking

Adults, 18–70+	Vitamin D	Placebo	Hypertension/blood pressure	Minimum 12 months follow-up in cohort studies. Minimum 4-week intervention in intervention studies	Relevant for the general population in the Nordic and Baltic countries	RCTs, cohort studies and case–control studies	Low	New SR are published, but hypertension/blood pressure was included in NNR2012
Adults	Plasma 25(OH), vitamin D	Dose-response	Vitamin D sufficiency (total mortality and bone health)	Minimum 4-week intervention in intervention studies	Relevant for the general population in the Nordic and Baltic countries	Interventions and mendelian randomisation studies	High	Appropriate cut-of values for sufficiency essential for setting DRVs. Several new large cohort and clinical studies, including Mendelian randomisation

Fat and fatty acids								

Population	Intervention or exposure	Comparators	Outcomes	Timing	Setting	Study design	Ranking	Argument for ranking

Adult population	Omega-3 fatty acids	Low versus high	Type 2 diabetes	Minimum of 2 years	Nordic, high-income countries	Controlled trials and cohort studies	High	Important public health issue. New data have emerged
Adults and elderly population	Quality of fat	Low versus high	Mental/brain health/cognition	Minimum of 2 years	Nordic, high-income countries	Cohort studies	High	Important public health issue. New data have emerged

Sodium								

Population	Intervention or exposure	Comparators	Outcomes	Timing	Setting	Study design	Ranking	Argument for ranking

Adults	Sodium intake	Low versus high, dose response to find protective level	Risk of CVD and indicators of CVD	Minimum 4-week intervention in intervention studies, Minimum 12 months follow-up in cohort studies	Relevant for the general population in the Nordic and Baltic countries	Prospective cohort studies and intervention	Low	The topic has been addressed by qSR

Ultra-processed foods								

Population	Intervention or exposure	Comparators	Outcomes	Timing	Setting	Study design	Ranking	Argument for ranking

All groups: pregnant, children, adolescents and adults	Degree of ultra-processed foods in the diet	No/low intake versus high intake of ultraprocessed foods (UPFs)	Noncommunicable diseases (NCDs) Mortality	Minimum 12 months follow-up in cohort studies	Relevant for the general population in the Nordic and Baltic countries	Prospective studies	High	High public interest and media attention

Meat								

Population	Intervention or exposure	Comparators	Outcomes	Timing	Setting	Study design	Ranking	Argument for ranking

Adult participants in the various cohorts included in the SRs	Meat (processed or unprocessed red meat) White meat	No or low consumption versus high consumption	All-cause mortality CVD and diabetes	Minimum 12 months follow-up in for prospective studies and 1 month for RCTs	Relevant for the general population in the Nordic and Baltic countries	Prospective cohort studies	High	High public interest and media attention, especially connected to sustainability issues

Fats and oils								

Population	Intervention or exposure	Comparators	Outcomes	Timing	Setting	Study design	Ranking	Argument for ranking

Adults, 18–70+ years	Vegetable oils (olive, sunflower and rapeseeds), and palm and coconut oils	Different consumption levels	Mortality, CVD, T2D and cancer	Minimum 12 months follow-up in cohort studies. Minimum 4-week intervention in intervention studies	Relevant for the general population in the Nordic and Baltic countries	RCTs and cohort studies	Medium	Establishing possible benefits of rapeseed oil would be important in the Nordic food environment. However, focusing on fatty acid level might be of greater importance
Children and adults, 1–70+ years	Vegetable oils (olive, sunflower and rapeseeds), and palm and coconut oils	Different consumption levels	Blood lipids	Minimum 12 months follow-up in cohort studies. Minimum 4-week intervention in intervention studies	Relevant for the general population in the Nordic and Baltic countries	RCTs, cohort studies, c-c studies and cross-sectional studies	Medium	
Children and adults, 1–70+ years	Vegetable oils (olive, sunflower and rapeseeds), and palm and coconut oils	Different consumption levels	Overweight and obesity	Minimum 12 months follow-up in cohort studies. Minimum 4-week intervention in intervention studies	Relevant for the general population in the Nordic and Baltic countries	RCTs, cohort studies, c-c studies and cross-sectional studies	Medium	

Calcium								

Population	Intervention or exposure	Comparators	Outcomes	Timing	Setting	Study design	Ranking	Argument for ranking

Healthy pregnant women and their offspring	Ca exposure: supplement + diet	Different levels of exposures Confounders: supplemental exposure of other nutrients and energy intake	Mother: hypertensive disorders, pre-eclampsia and preterm birth Offspring: birth weight and BP level	Minimum 12 months follow-up in cohort studies. Minimum 4-week intervention in intervention studies	Primary health care	RCTs, cohort studies and c-c studies	High	Common outcome in Nordic countries. Ongoing shift to more plant-based diets might add to the need for supplementation
Adult population/men, 50 years + older	Ca exposure: supplement + diet	Different levels of exposures	Colorectal cancer and prostate cancer	Minimum 12 months follow-up in cohort studies. Minimum 4-week intervention in intervention studies	Relevant for the general population in the Nordic and Baltic countries	RCTs, cohort studies and c-c studies	Low	The topic is currently addressed through other qSRs

Calcium								

Population	Intervention or exposure	Comparators	Outcomes	Timing	Setting	Study design	Ranking	Argument for ranking

Adult population, 50 years + older	Ca exposure: supplement + diet	Different levels of exposures Confounders: supplemental exposure of vitamin D	Injurious falls and fractures	Minimum 12 months follow-up in cohort studies. Minimum 4-week intervention in intervention studies	Relevant for the general population in the Nordic and Baltic countries	RCTs and cohort studies	Low	The topic is currently addressed through other qSRs

B12								

Population	Intervention or exposure	Comparators	Outcomes	Timing	Setting	Study design		

Healthy pregnant women	B12 exposure: supplement and diet B12 status	Different level of exposures	Preterm birth Low birth weight	Minimum 12 months follow-up in cohort studies. Minimum 4-week intervention in intervention studies	Primary health care	RCTs, cohort studies and c-c studies	High	B12 insufficiency during pregnancy is common even in non-vegetarian population
Elderly, 60 years and older	B12 exposure: supplement and diet B12 status	Different level of exposures	Neurological functions: cognitive decline and dementia	Minimum 12 months follow-up in cohort studies. Minimum 4-week intervention in intervention studies	Relevant for the general population in the Nordic and Baltic countries	RCTs, cohort studies, c-c studies and cross-sectional studies	Medium	Findings somewhat conflicting and partly shown only with newer biomarkers
Whole population, lifespan approach and all age groups	B12 exposure: supplement and dietary intakes in different diets: vegetarian, vegan and omnivore	Different level of exposures	B12 status in different age groups	Minimum 12 months follow-up in cohort studies. Minimum 4-week intervention in intervention studies	Relevant for the general population in the Nordic and Baltic countries	RCTs, cohort studies, c-c studies and cross-sectional studies	High	New relevant data available (from RCTs in Nordic countries as well)
Children following vegan diet (public call)	B12 exposure: supplement and fortified foods	Different level of exposures	B12 requirement to defend deficiency and to maintain normal function	Minimum 12 months follow-up in cohort studies. Minimum 4-week intervention in intervention studies	Relevant for the general population in the Nordic and Baltic countries	RCTs, cohort studies, c-c studies and cross-sectional studies	Medium	Important topic. However, the SR may lack well conducted studies to be based on

Biotin								

Population	Intervention or exposure	Comparators	Outcomes	Timing	Setting	Study design	Ranking	Argument for ranking

Healthy and pregnant and lactating women	Biotin: intake, status propionyl-CoA carboxylase (PCC), pyruvate carboxylase (PC), acetyl-CoA carboxylase (ACC) and deficiency (3HIA and 3 HIA-carnitine)	Different levels of exposures	Clinical abnormalities in offspring: *growth, retardation, congenital malformation, neurological disorders, dermatological abnormalities; genome stability (genomic damage in lymphocytes)*	Minimum 12 months follow-up in cohort studies. Minimum 4-week intervention in intervention studies	Primary health care	Prospective birth cohorts, RCTs and cross-sectional studies	Low	We need more data in order to do a SR. Not enough literature. New evidence unlikely to influence DRV

Fish, fish products and seafood								

Population	Intervention or exposure	Comparators	Outcomes	Timing	Setting	Study design	Ranking	Argument for ranking

Women and their offspring	n-3 LPUFAs from fish or supplementation	Supplementation versus placebo (in RCTs) OR above versus below NNR2012 recommendations	Asthma and allergies in the offspring	Minimum 12 months follow-up in cohort studies. Minimum 4-week intervention in intervention studies	Relevant for the general population in the Nordic and Baltic countries	RCTs and observational studies	High	New relevant data available

Nuts								

Population	Intervention or exposure	Comparators	Outcomes	Timing	Setting	Study design	Ranking	Argument for ranking

Adults, 18–75 years	Nuts intake higher than current, for example, 30 g/day	High versus low intake	CVD (or other heart outcome?)	Minimum 12 months follow-up in cohort studies. Minimum 4-week intervention in intervention studies	Relevant for the general population in the Nordic and Baltic countries	RCTs, cohort studies and case–control studies	High	Very little info on nuts in NNR2012. New relevant data available

Milk and dairy								

Population	Intervention or exposure	Comparators	Outcomes	Timing	Setting	Study design	Ranking	Argument for ranking

The general population, adults 18–80 years	Full fat dairy	Low fat dairy	CVD and blood lipids	Minimum 12 months follow-up in cohort studies. Minimum 4-week intervention in intervention studies	Nordic, other EU or US population	Intervention studies and observational studies	Medium	Findings published since 2012 provide no consistent evidence that could challenge those previous conclusions on DRVs or FBGDs from NNR 2012

Micronutrients								

Population	Intervention or exposure	Comparators	Outcomes	Timing	Setting	Study design	Ranking	Argument for ranking

Adults	Micronutrient status (or intake)	Deficiency, sufficiency and excess	COVID-19 infection and severity	Minimum 12 months follow-up in cohort studies. Minimum 4-week intervention in intervention studies	General population relevant for Nordic and Baltic countries	Prospective cohort studies and interventions	High	Many nutrients have powerful immunomodulatory actions with the potential to alter susceptibility to COVID-19 infection, progression to symptoms, likelihood of severe disease and survival

* ROB-Nobs, Risk of bias for nutrition observational studies tool: ‘low’, ‘moderate’, ‘serious’, ‘critical’ or ‘no information’.

The table contains all shortlisted topics from the 51 ScRs.

## Step 5. The grading of SR topics into high, medium and low importance

Subsequently, the NNR2022 Committee members graded individually the PI/ECOTSS into ‘High’ (*n* = 21), ‘Medium’ (*n* = 9) or ‘Low’ (*n* = 22) importance (Table 3), based on the criteria described (Box 1). The final grading was then decided in a consensus process. This process took more than 6 months and included careful evaluation of all the 51 ScRs as well as secondary literature- and citation searches.

## Step 6. The ranking of SR topics of high importance

The ranking of PI/ECOTSS statements with high importance was performed in a modified Delphi process amongst the NNR2022 Committee members. The Delphi process is a general, structured, interactive technique involving a panel of experts. It can also include face-to-face meetings. Delphi is based on the principle that decisions from a structured group of individuals are more accurate than those from unstructured groups. The experts answer questionnaires in two or more rounds. After each round, a facilitator provides an anonymised summary of the experts’ voting from the previous round as well as the reasons they provided for their judgments. Thus, experts are encouraged to revise their earlier answers in light of the replies of other members of their panel. It is assumed that during this process, the range of the answers will decrease, and the group will converge towards a consensus (16).

The NNR2022 Committee individually prioritised the 21 PI/ECOTSS statements graded ‘High importance’ by giving each PI/ECOTSS statement a priority between 1 and 21.

An anonymised summary table, including arguments for prioritisation, was presented for the whole Committee by the NNR2022 project secretary. The Committee members were encouraged to revise their initial prioritisations in light of the discussion in the Committee meetings. A new anonymised summary table was then presented to the whole Committee in the next meeting. This procedure was repeated three times before a consensus was reached. The ranked list of the SR topics, and the main arguments for ranking, is presented in Table 4. The formulation of the PI/ECOTSS was adjusted during the prioritisation process; thus, the formulation of the PI/ECOTSS in Table 4 is more specific compared with Table 3.

**Table 4:** Prioritised topics for systematic reviews.

Topic								
Protein								
Population	Intervention or exposure	Comparators	Outcomes	Timing	Setting	Study design	Ranking	Argument for ranking
Children (4 months to 5 years)	Total protein intake Amount and different sources of protein, that is, plant versus animal protein intake	Highest versus lowest protein intakes as defined by, for example, quartiles or risk difference per gram protein from one source relative to other sources. Comparison of various protein intakes in RCTs	Growth/anthropometric outcomes: weight (kg or *z*-scores/standard deviation scores (SDS)), length (cm or *z*-scores/SDS) and BMI (absolute measures or *z*-scores). Risk of overweight/obesity. Body composition (indices, e.g. fat free mass (FFM), fat mass (FM)	Minimum 6 months follow-up in cohort studies. Minimum 4 weeks intervention in intervention studies (depending on the age of the child)	Relevant for Nordic setting (excludes, for example, populations with high prevalence of childhood malnutrition)	Randomised and non-randomised controlled intervention studies. Prospective cohort studies, nested case–control and case–cohort studies	1	Several high-quality studies published since NNR2012. Evidence may be stronger than concluded in NNR2012. The reasons why existing SRs produce different results should be explored. More thorough assessment can be made. Many SRs did not include animal versus plant protein

Pulses and legumes								

Population	Intervention or exposure	Comparators	Outcomes	Timing	Setting	Study design	Ranking	Argument for ranking

Adults (≥18 years)	Pulses/legumes (subgroups if possible), exclude peanuts	No/low versus high consumption Dose-response	Atherosclerotic cardiovascular disease mortality and morbidity (total and subgroups) and type-2 diabetes in prospective studies CVD qualified surrogate endpoints and diabetes/insulin resistance/sensitivity in interventions	Minimum 12 months for prospective studies, 1 month for RCTs, depending on outcome	Relevant for the general population in the Nordic and Baltic countries	Prospective cohort studies and interventions	2	High priority due to focus on sustainability of diets and not covered by NNR2012. Increasing consumption, greater variety and several recent high-quality studies. Important to appraise this association since these foods are important as substitutes for meat. Overview of health effects of different kinds of pulses would be valuable for setting FBDGs

Protein								

Population	Intervention or exposure	Comparators	Outcomes	Timing	Setting	Study design	Ranking	Argument for ranking

Adults	Plant protein intake	Animal protein intake	Atherosclerotic, cardiovascular disease, mortality and morbidity (total and subgroups) and type-2 diabetes in prospective studies. CVD qualified surrogate endpoints and diabetes/insulin resistance/sensitivity in RCTs	Minimum 12 months follow-up in cohort studies. Minimum 4 weeks intervention in intervention studies	Relevant for the general population in the Nordic and Baltic countries	RCT and prospective cohorts	3	Relevant for our encouragement to eat more plant based Important to summarise the new evidence for replacing animal-based protein with plant-based protein in relation to most common chronic diseases in Nordic countries. New RCTs available also from Nordic countries. Relevant for recommendation on protein and on FBDGs. New literature is available. Increasing consumption in Nordic countries.

Vitamin B_12_								

Population	Intervention or exposure	Comparators	Outcomes	Timing	Setting	Study design	Ranking	Argument for ranking

Susceptible groups, that is: 1) children (0–18 years), 2) young adults (18–35 years), 3) pregnant and 4) lactating women, 5) older adults (≥65 years) and 6) vegetarians including vegans	B_12_ exposure: supplemental and dietary intake	Different level of exposures	B_12_ status: * s/p- B12 *s/p- HOLO-TC *s/p-MMA *s/p-tHcy *Combined indicators *Breastmilk B^12^ (relevant in infants)	Minimum 12 months follow-up in cohort studies. Minimum 4 weeks intervention in intervention studies	Relevant for the general population in the Nordic and Baltic countries	RCTs, cohort studies, case–control studies, cross-sectional studies (the last one relevant for limited periods as pregnancy and lactation)	4	High priority due to focus on sustainability of diets and might affect DRVs. In the context of a more plant-bases diet, it is important to know how B12 status is impacted in the most vulnerable groups. This SR would identify data that facilitates setting DRVs for vulnerable groups

Fat and fatty acids								

Population	Intervention or exposure	Comparators	Outcomes	Timing	Setting	Study design	Ranking	Argument for ranking

Adults (≥50 years)	Quality of fat (e.g. E% from different subtypes, such as saturated fatty acids (SFA), monounsaturated fatty acids (MUFA), polyunsaturated farry acids (PUFA)not total amount)	Other level of intake and substitution models	Outcome: Specific dementias: Alzheimer’s disease (ICD8 290.10 and ICD10 F00 and G30), vascular dementia (ICD10 F01) and unspecified dementia (ICD8 290.18 and ICD10). All-cause dementia. For intervention studies: mild cognitive impairment (G31) and cognitive decline	Minimum 5 years follow-up in cohort studies. Minimum 12 months intervention in intervention studies. The duration of follow-up depends on age at inclusion	Relevant for the general population in the Nordic and Baltic countries	Prospective cohort studies and intervention studies	5	High priority due to new evidence on outcome. With ageing population and increasing prevalence of cognitive disorders this is important, health issues and relationship unclear. Increasing elderly population justifies at least one topic on this group

Meat and meat products								

Population	Intervention or exposure	Comparators	Outcomes	Timing	Setting	Study design	Ranking	Argument for ranking

Adults	White meat	No or low consumption versus high consumption, white meat replaced other red meat	All-cause mortality, CVD and type 2 diabetes and risk factors for the diseases in RCTs	Minimum 12 months follow-up for prospective studies and 1 month for RCTs	Relevant for the general population in the Nordic and Baltic countries	Prospective cohort studies and RCTs	6	High priority due to focus on environmental sustainability and more focus on a plant-based diet. High relevance in the Nordic and Baltic countries. Important to determine the effects of white meat consumption

Fish and fish products								

Population	Intervention or exposure	Comparators	Outcomes	Timing	Setting	Study design	Ranking	Argument for ranking

Women and their offspring	n-3 LPUFAs from supplements	Supplementation versus placebo (in RCTs)	Asthma and allergies in the offspring	Minimum 4 weeks intervention in intervention studies	Relevant for the general population in the Nordic and Baltic countries	RCTs	7	High priority due to the prevalence of asthma and allergies. Important to document the effect due to in context of recommendations of a more plant-based diet

Nuts								

Population	Intervention or exposure	Comparators	Outcomes	Timing	Setting	Study design	Ranking	Argument for ranking

Adults	Nuts intake higher than current, for example, 30 g/day	High versus low intake	CVD and T2D in observational studies AND intermediate endpoints for CVD in RCTs	Minimum 12 months follow-up in cohort studies. Minimum 4 weeks intervention in intervention studies	Relevant for the general population in the Nordic and Baltic countries	RCTs, cohort studies and case–control studies	8	High priority due to focus on environmental sustainability and shift towards a more plant-based diet. Evidence needed to establish FBDGs

Dietary fibre								

Population	Intervention or exposure	Comparators	Outcomes	Timing	Setting	Study design	Ranking	Argument for ranking

Children	Dietary fibre and its subgroupings, for example, soluble and in-soluble. Or subgroups related to the fractions in chemical analysis. Or depending on origin (grain, pulses, vegetables and fruits)	High and low dose-response	Bowel function Energy availability. Nutrient availability. All including risks of high intake.	Short time/few days of follow-up, depending on study design and outcome	Relevant for the general population in the Nordic and Baltic countries	Prospective cohort studies and RCTs	9	High priority due to relevance for the Nordic and Baltic populations

**Fig. 2:**
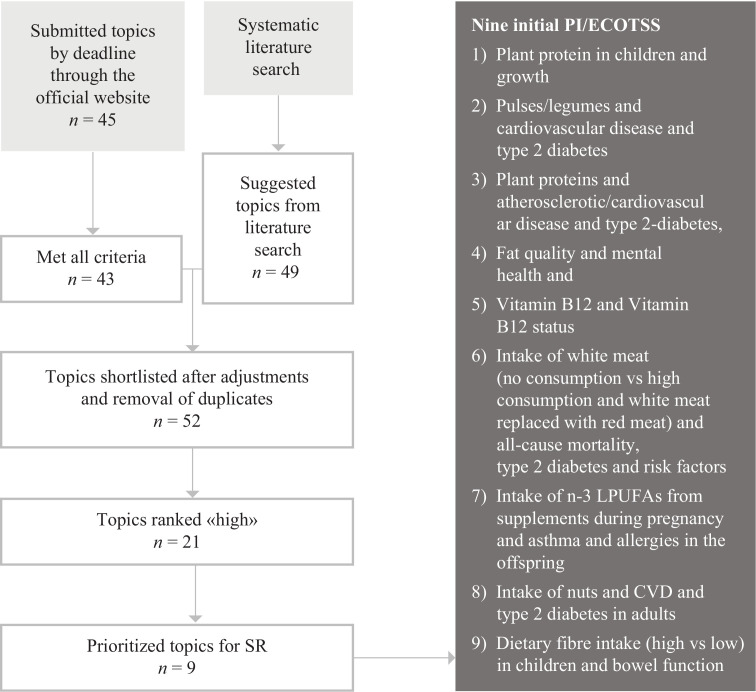
Screening and prioritisation of topics from public call and scoping reviews.

The first five top prioritised topics, as well as all relevant background documentation, was submitted to the NNR SR Centre for their comments. In a dialog between the NNR SR Centre and the NNR2022 Committee, the final PI/ECOTSS statements for the five prioritised topics were formulated and agreed on by January 13, 2021 (Table 4). The four remaining PI/ECOTSS statements was agreed on in June 2021. Results from step 1 to 6 in the procedure are summarised in Fig. 2.

A protocol (17–21) will be developed for all *de novo* SRs by the SR Centre and published in PROSPERO (https://www.crd.york.ac.uk/prospero/). The NNR2022 Committee and the topic experts (i.e. the scientists recruited to author the respective nutrient or food group chapters in NNR2022) will be consulted when finalising the protocols.

## Discussion

Given the extent of scientific publications in the field of nutrition and health, and the limited resources available to summarise present research status rigorously and transparently, we have developed a procedure for prioritisation of topics that may be selected for SRs. The selection of topics for *de novo* SRs is central in the NNR2022 project, as the results of these SRs may cause adjustment of existing DRVs and FBDGs. That is why we have developed this extensive process for prioritisation of SR topics. The current paper describes the results of this procedure used to prioritise topics for *de novo* SRs in the NNR2022 project. The nine prioritised PI/ECOTSS statements include the following exposure–outcome pairs: 1) plant protein intake in children and growth, 2) pulses/legumes, and cardiovascular disease and type 2 diabetes, 3) plant proteins, and atherosclerotic/cardiovascular disease and type 2 diabetes, 4) fat quality and mental health and 5) vitamin B^12^ and vitamin B^12^ status, 6) intake of white meat (no consumption vs. high consumption and white meat replaced with red meat), and all-cause mortality, type 2 diabetes and risk factors, 7) intake of n-3 LPUFAs from supplements during pregnancy and asthma and allergies in the offspring, 8) nuts intake, and CVD and type 2 diabetes in adults, 9) dietary fibre intake (high vs. low) in children and bowel function (Table 4). Small adjustments of the PI/ECOTSS may occur during the development of the protocols. The final wording will be available in the published protocols.

The nine top SR topics are given high priority since significant new evidence within these topics might change the current recommendations. Additionally, increased adherence and more focus on plant-based diets and an environmentally sustainable diet were also important arguments for several of the SR priorities. Health effects of such changes must be considered and evaluated before potentially adjusting DRVs and FBDGs. The topic on vitamin B^12^ status is also partly due to the aging population and related health consequences. The rational for the prioritisations is given in Table 4.

A delicate balance must be considered when PI/ECOTSS statements are formulated. They may be too narrow to be generalisable. Additionally, it is always tempting to broaden the scope, for example, the exposure, the population or the outcome, but this may massively influence the resources needed for performing the SR. Too broad PI/ECOTSS statements may also be more imprecise and mask specific questions. In this process, we have tried, openly and explicitly, to identify the most relevant PI/ECOTSS for adjusting DRVs and FBDGs in the Nordic and Baltic countries, but, at the same time, use the limited resources available in the most cost-effective manner.

Traditionally, the working group responsible for developing national DRVs and FBDGs select SR topics based on their own scientific knowledge and after consultation with appointed scientists in the field of interest. In the NNR2022 project, we have involved numerous scientists, health professionals, national food and health authorities, food manufacturers, other stakeholders and the general population to generate a large and representative pool of potential SR topics. This pool of topics was valuable when the NNR2022 Committee performed the prioritisation process in the modified Delphi process. Selection of SR topics can never be a fully objective exercise. Some stakeholders may be more proactive than others. The NNR2022 Committee tried to use all available information, independent of subjective engagement by stakeholders. In the end, selection of SR topics was the decision of the NNR2022 Committee.

Although consensus was reached in the NNR2022 Committee, it does not necessarily mean that we have concluded with the ‘correct’ selection. Several other topics might have been considered and prioritised. The question about what is most important in nutritional sciences is large and open. In the present project, we have, however, focused on topics with substantial recent data and public health concern, which is most relevant for setting DRVs and FBDGs in the Nordic and Baltic countries.

A limitation of our study is the literature search (Supplementary Table 2) used to develop the 51 ScRs. We decided initially to limit the search to reviews published in 2011 and later with the filter ‘Humans’. If the search resulted in ≥500 items, we limited the search to papers with the nutrients or food groups in the title. If still ≥500 items, we included the additional requirements: ‘Diet’ OR ‘Dietary’ OR ‘FOOD’ OR ‘Nutrition’ OR ‘Nutritional’. If still ≥500 items, we limited the search to only include ‘Systematic reviews’. The reason why we initially selected to search for reviews published after 2010 is that it is likely that a topic with significant new and relevant data would have been discussed in a review paper published after the search date in the previous edition of NNR. In this type of strategy, we omit all original publications. However, DRVs or FBDGs are seldom, or never, revised based on one or a few original publications. In the present literature search process to identify SR topics, only original study results found important enough to be cited and discussed in review papers are candidate for SR topics.

Additionally, if a large number of reviews were identified for a single nutrient or food group (i.e. ≥500 papers), we added sequentially additional relevant limitations, simply to reduce the burden of the authors of the 51 ScRs. In total, 13,992 reviews were identified and scrutinised by the ScR authors. Although we do not believe that other topics would have been prioritised with an even more comprehensive search strategy, we cannot rule out the possibility that some important topics have been missed.

It is important to note that the present literature search was only used to select topics for *de novo* SRs. In each of the 51 nutrient and food group chapters that will be part of the final NNR2022 report, a separate literature search will be performed and described.

The organisation, the principles and the methodologies developed in the NNR2022 project build on processes similar to other national authorities or international health organisations. The procedure described in this paper, together with the three previous principle and methodology papers from the NNR2022 project (2–4), may serve as a framework that other national health authorities or organisations can adapt when developing national DRVs and FBDGs.

A large amount of resources and extensive interdisciplinary front-edge competence is needed to develop national DRVs and FBDGs. No or few single nations have these qualifications alone. Thus, international collaboration and global harmonisation of methodological approaches are highly needed. The NNR2022 project, which is a collaboration between the food and health authorities in Denmark, Estonia, Finland, Iceland, Latvia, Lithuania, Norway and Sweden, represents such an international effort for harmonisation and sharing of resources and competence.

## Summary and conclusions

SRs are the preferred method to summarise the causal relationship between nutrient or food group exposure and a health outcome. They are the main fundament for developing DRVs and FBDGs. In this paper, we describe the results of an open, transparent six-step procedure to identify and prioritise topics most appropriate for *de novo* SRs in the NNR2022 project. The nine prioritised PI/ECOTSS include the following exposure–outcome pairs: 1) plant protein intake in children and body growth, 2) pulses/legumes intake, and cardiovascular disease and type 2 diabetes, 3) plant protein intake in adults, and atherosclerotic/cardiovascular disease and type 2 diabetes, 4) fat quality and mental health, 5) vitamin B^12^ and vitamin B^12^ status, 6) intake of white meat (no consumption vs. high consumption and white meat replaced with red meat), and all-cause mortality, type 2 diabetes and risk factors, 7) intake of n-3 LPUFAs from supplements during pregnancy and asthma and allergies in the offspring, 8) nuts intake, and CVD and type 2 diabetes in adults, 9) dietary fibre intake (high vs. low) in children and bowel function.

## Supplementary Material

The Nordic Nutrition Recommendations 2022 – prioritisation of topics for *de novo* systematic reviews
