# Supplementary material for: The Nordic Nutrition Recommendations 2022 – prioritisation of topics for de novo systematic reviews
Source: Food Nutr Res. 2021 Oct 8;65:10.29219/fnr.v65.7828. doi: 10.29219/fnr.v65.7828 (PMC8897982; doi:10.29219/fnr.v65.7828)
Supplement: The Nordic Nutrition Recommendations 2022 – prioritisation of topics for de novo systematic reviews [file FNR-65-7828-s001.docx]

**Supplementary table 2: Scoping Reviews search strategy**

1. Topic
2. Publication date 2011 to present
3. Filter: Humans
4. Publication Type: Review

If ≥500 items:

Limit Topic to title, e.g.: *Topic*[TI]

If still ≥500 items:

Add (Diet OR Dietary OR FOOD OR Nutrition OR Nutritional)

If still ≥500 items:

Limit [Publication Type] to Systematic review

**Nutrients**

| Topic | No. items | Query with link |
| --- | --- | --- |
| Energy | [148](https://www.ncbi.nlm.nih.gov/pubmed/?term=(((((energy%5BTitle%5D))+AND+systematic+review%5BPublication+Type%5D)+AND+(%222011%22%5BDate+-+Publication%5D+%3A+%223000%22%5BDate+-+Publication%5D)))+AND+Humans%5BFilter%5D) (17/09/2019) | [energy[Title] AND systematic review[Publication Type] AND ("2011"[Date - Publication] : "3000"[Date - Publication]) AND Humans[Filter]](https://www.ncbi.nlm.nih.gov/pubmed/?term=(((((energy%5BTitle%5D))+AND+systematic+review%5BPublication+Type%5D)+AND+(%222011%22%5BDate+-+Publication%5D+%3A+%223000%22%5BDate+-+Publication%5D)))+AND+Humans%5BFilter%5D) |
| Fat and fatty acids | [192](https://www.ncbi.nlm.nih.gov/pubmed?term=%28%28%28%28fat%5BTitle%5D%20OR%20%22fatty%20acids%22%5BTitle%5D%29%20AND%20systematic%20review%5BPublication%20Type%5D%29%20AND%20humans%5BFilter%5D%29%20AND%20%28%222011%22%5BPDAT%5D%20%3A%20%223000%22%5BPDAT%5D%29%29%20AND%20%28%28%22diet%22%5BMeSH%20Terms%5D%20OR%20%22diet%22%5BAll%20Fields%5D%29%20OR%20%28%22diet%22%5BMeSH%20Terms%5D%20OR%20%22diet%22%5BAll%20Fields%5D%20OR%20%22dietary%22%5BAll%20Fields%5D%29%20OR%20%28%22food%22%5BMeSH%20Terms%5D%20OR%20%22food%22%5BAll%20Fields%5D%29%20OR%20%28%22nutritional%20status%22%5BMeSH%20Terms%5D%20OR%20%28%22nutritional%22%5BAll%20Fields%5D%20AND%20%22status%22%5BAll%20Fields%5D%29%20OR%20%22nutritional%20status%22%5BAll%20Fields%5D%20OR%20%22nutrition%22%5BAll%20Fields%5D%20OR%20%22nutritional%20sciences%22%5BMeSH%20Terms%5D%20OR%20%28%22nutritional%22%5BAll%20Fields%5D%20AND%20%22sciences%22%5BAll%20Fields%5D%29%20OR%20%22nutritional%20sciences%22%5BAll%20Fields%5D%29%20OR%20Nutritional%5BAll%20Fields%5D%29&cmd=DetailsSearch) (17/09/2019) | [(fat[Title] OR "fatty acids"[Title]) AND systematic review[Publication Type] AND humans[Filter] AND ("2011"[Date - Publication] : "3000"[Date - Publication]) AND (Diet OR Dietary OR Food OR Nutrition OR Nutritional)](https://www.ncbi.nlm.nih.gov/pubmed?term=%28%28%28%28fat%5BTitle%5D%20OR%20%22fatty%20acids%22%5BTitle%5D%29%20AND%20systematic%20review%5BPublication%20Type%5D%29%20AND%20humans%5BFilter%5D%29%20AND%20%28%222011%22%5BPDAT%5D%20%3A%20%223000%22%5BPDAT%5D%29%29%20AND%20%28%28%22diet%22%5BMeSH%20Terms%5D%20OR%20%22diet%22%5BAll%20Fields%5D%29%20OR%20%28%22diet%22%5BMeSH%20Terms%5D%20OR%20%22diet%22%5BAll%20Fields%5D%20OR%20%22dietary%22%5BAll%20Fields%5D%29%20OR%20%28%22food%22%5BMeSH%20Terms%5D%20OR%20%22food%22%5BAll%20Fields%5D%29%20OR%20%28%22nutritional%20status%22%5BMeSH%20Terms%5D%20OR%20%28%22nutritional%22%5BAll%20Fields%5D%20AND%20%22status%22%5BAll%20Fields%5D%29%20OR%20%22nutritional%20status%22%5BAll%20Fields%5D%20OR%20%22nutrition%22%5BAll%20Fields%5D%20OR%20%22nutritional%20sciences%22%5BMeSH%20Terms%5D%20OR%20%28%22nutritional%22%5BAll%20Fields%5D%20AND%20%22sciences%22%5BAll%20Fields%5D%29%20OR%20%22nutritional%20sciences%22%5BAll%20Fields%5D%29%20OR%20Nutritional%5BAll%20Fields%5D%29&cmd=DetailsSearch) |
| Carbohydrates | [238](https://www.ncbi.nlm.nih.gov/pubmed?term=%28%28%28carbohydrate%5BTitle%5D%20OR%20carbohydrate%27%5BTitle%5D%20OR%20carbohydrate%27s%5BTitle%5D%20OR%20carbohydrateassociated%5BTitle%5D%20OR%20carbohydratecontaining%5BTitle%5D%20OR%20carbohydrated%5BTitle%5D%20OR%20carbohydratedeficient%5BTitle%5D%20OR%20carbohydratelectin%5BTitle%5D%20OR%20carbohydratemetabolizing%5BTitle%5D%20OR%20carbohydratephosphate%5BTitle%5D%20OR%20carbohydratephosphorus%5BTitle%5D%20OR%20carbohydrateprotein%5BTitle%5D%20OR%20carbohydrates%5BTitle%5D%20OR%20carbohydrates%27%5BTitle%5D%20OR%20carbohydratesan%5BTitle%5D%20OR%20carbohydratesubstrates%5BTitle%5D%20OR%20carbohydratex%5BTitle%5D%29%20AND%20%28review%5BPublication%20Type%5D%20AND%20%28%222011%22%5BPDAT%5D%20%3A%20%223000%22%5BPDAT%5D%29%29%29%20AND%20Humans%5BFilter%5D%29%20AND%20%28%28%22diet%22%5BMeSH%20Terms%5D%20OR%20%22diet%22%5BAll%20Fields%5D%29%20OR%20%28%22diet%22%5BMeSH%20Terms%5D%20OR%20%22diet%22%5BAll%20Fields%5D%20OR%20%22dietary%22%5BAll%20Fields%5D%29%20OR%20%28%22food%22%5BMeSH%20Terms%5D%20OR%20%22food%22%5BAll%20Fields%5D%29%20OR%20%28%22nutritional%20status%22%5BMeSH%20Terms%5D%20OR%20%28%22nutritional%22%5BAll%20Fields%5D%20AND%20%22status%22%5BAll%20Fields%5D%29%20OR%20%22nutritional%20status%22%5BAll%20Fields%5D%20OR%20%22nutrition%22%5BAll%20Fields%5D%20OR%20%22nutritional%20sciences%22%5BMeSH%20Terms%5D%20OR%20%28%22nutritional%22%5BAll%20Fields%5D%20AND%20%22sciences%22%5BAll%20Fields%5D%29%20OR%20%22nutritional%20sciences%22%5BAll%20Fields%5D%29%20OR%20Nutritional%5BAll%20Fields%5D%29&cmd=DetailsSearch) (17/09/2019) | [carbohydrate*[Title] AND review[Publication Type] AND ("2011"[Date - Publication] : "3000"[Date - Publication]) AND Humans[Filter] AND (Diet OR Dietary OR Food OR Nutrition OR Nutritional)](https://www.ncbi.nlm.nih.gov/pubmed?term=%28%28%28carbohydrate%5BTitle%5D%20OR%20carbohydrate%27%5BTitle%5D%20OR%20carbohydrate%27s%5BTitle%5D%20OR%20carbohydrateassociated%5BTitle%5D%20OR%20carbohydratecontaining%5BTitle%5D%20OR%20carbohydrated%5BTitle%5D%20OR%20carbohydratedeficient%5BTitle%5D%20OR%20carbohydratelectin%5BTitle%5D%20OR%20carbohydratemetabolizing%5BTitle%5D%20OR%20carbohydratephosphate%5BTitle%5D%20OR%20carbohydratephosphorus%5BTitle%5D%20OR%20carbohydrateprotein%5BTitle%5D%20OR%20carbohydrates%5BTitle%5D%20OR%20carbohydrates%27%5BTitle%5D%20OR%20carbohydratesan%5BTitle%5D%20OR%20carbohydratesubstrates%5BTitle%5D%20OR%20carbohydratex%5BTitle%5D%29%20AND%20%28review%5BPublication%20Type%5D%20AND%20%28%222011%22%5BPDAT%5D%20%3A%20%223000%22%5BPDAT%5D%29%29%29%20AND%20Humans%5BFilter%5D%29%20AND%20%28%28%22diet%22%5BMeSH%20Terms%5D%20OR%20%22diet%22%5BAll%20Fields%5D%29%20OR%20%28%22diet%22%5BMeSH%20Terms%5D%20OR%20%22diet%22%5BAll%20Fields%5D%20OR%20%22dietary%22%5BAll%20Fields%5D%29%20OR%20%28%22food%22%5BMeSH%20Terms%5D%20OR%20%22food%22%5BAll%20Fields%5D%29%20OR%20%28%22nutritional%20status%22%5BMeSH%20Terms%5D%20OR%20%28%22nutritional%22%5BAll%20Fields%5D%20AND%20%22status%22%5BAll%20Fields%5D%29%20OR%20%22nutritional%20status%22%5BAll%20Fields%5D%20OR%20%22nutrition%22%5BAll%20Fields%5D%20OR%20%22nutritional%20sciences%22%5BMeSH%20Terms%5D%20OR%20%28%22nutritional%22%5BAll%20Fields%5D%20AND%20%22sciences%22%5BAll%20Fields%5D%29%20OR%20%22nutritional%20sciences%22%5BAll%20Fields%5D%29%20OR%20Nutritional%5BAll%20Fields%5D%29&cmd=DetailsSearch) |
| Dietary fibre | 153 (17/02/2020) | [("fiber"[Title] OR "fibre"[Title] AND diet*) AND ("2011"[Date - Publication] : "3000"[Date - Publication]) AND Humans[Filter] AND Review[Publication Type]](https://pubmed.ncbi.nlm.nih.gov/?term=%28%22fiber%22%5BTitle%5D+OR+%22fibre%22%5BTitle%5D+AND+diet*%29+AND+%28%222011%22%5BDate+-+Publication%5D+%3A+%223000%22%5BDate+-+Publication%5D%29+AND+Humans%5BFilter%5D+AND+Review%5BPublication+Type%5D&sort=pubdate) |
| Protein | [112](https://www.ncbi.nlm.nih.gov/pubmed?term=%28%28protein%5BTitle%5D%20AND%20%28systematic%20review%5BPublication%20Type%5D%20AND%20%28%222011%22%5BPDAT%5D%20%3A%20%223000%22%5BPDAT%5D%29%29%29%20AND%20Humans%5BFilter%5D%29%20AND%20%28%28%22diet%22%5BMeSH%20Terms%5D%20OR%20%22diet%22%5BAll%20Fields%5D%29%20OR%20%28%22diet%22%5BMeSH%20Terms%5D%20OR%20%22diet%22%5BAll%20Fields%5D%20OR%20%22dietary%22%5BAll%20Fields%5D%29%20OR%20%28%22food%22%5BMeSH%20Terms%5D%20OR%20%22food%22%5BAll%20Fields%5D%29%20OR%20%28%22nutritional%20status%22%5BMeSH%20Terms%5D%20OR%20%28%22nutritional%22%5BAll%20Fields%5D%20AND%20%22status%22%5BAll%20Fields%5D%29%20OR%20%22nutritional%20status%22%5BAll%20Fields%5D%20OR%20%22nutrition%22%5BAll%20Fields%5D%20OR%20%22nutritional%20sciences%22%5BMeSH%20Terms%5D%20OR%20%28%22nutritional%22%5BAll%20Fields%5D%20AND%20%22sciences%22%5BAll%20Fields%5D%29%20OR%20%22nutritional%20sciences%22%5BAll%20Fields%5D%29%20OR%20Nutritional%5BAll%20Fields%5D%29&cmd=DetailsSearch) (17/09/2019) | [protein[Title] AND systematic review[Publication Type] AND ("2011"[PDAT] : "3000"[PDAT]) AND Humans[Filter] AND ("diet"[MeSH Terms] OR "diet"[All Fields] OR "diet"[MeSH Terms] OR "diet"[All Fields] OR "dietary"[All Fields] OR "food"[MeSH Terms] OR "food"[All Fields] OR "nutritional status"[MeSH Terms] OR "nutritional"[All Fields] AND "status"[All Fields] OR "nutritional status"[All Fields] OR "nutrition"[All Fields] OR "nutritional sciences"[MeSH Terms] OR "nutritional"[All Fields] AND "sciences"[All Fields] OR "nutritional sciences"[All Fields] OR Nutritional[All Fields])](https://www.ncbi.nlm.nih.gov/pubmed?term=%28%28protein%5BTitle%5D%20AND%20%28systematic%20review%5BPublication%20Type%5D%20AND%20%28%222011%22%5BPDAT%5D%20%3A%20%223000%22%5BPDAT%5D%29%29%29%20AND%20Humans%5BFilter%5D%29%20AND%20%28%28%22diet%22%5BMeSH%20Terms%5D%20OR%20%22diet%22%5BAll%20Fields%5D%29%20OR%20%28%22diet%22%5BMeSH%20Terms%5D%20OR%20%22diet%22%5BAll%20Fields%5D%20OR%20%22dietary%22%5BAll%20Fields%5D%29%20OR%20%28%22food%22%5BMeSH%20Terms%5D%20OR%20%22food%22%5BAll%20Fields%5D%29%20OR%20%28%22nutritional%20status%22%5BMeSH%20Terms%5D%20OR%20%28%22nutritional%22%5BAll%20Fields%5D%20AND%20%22status%22%5BAll%20Fields%5D%29%20OR%20%22nutritional%20status%22%5BAll%20Fields%5D%20OR%20%22nutrition%22%5BAll%20Fields%5D%20OR%20%22nutritional%20sciences%22%5BMeSH%20Terms%5D%20OR%20%28%22nutritional%22%5BAll%20Fields%5D%20AND%20%22sciences%22%5BAll%20Fields%5D%29%20OR%20%22nutritional%20sciences%22%5BAll%20Fields%5D%29%20OR%20Nutritional%5BAll%20Fields%5D%29&cmd=DetailsSearch) |
| Antioxidants | [309](https://www.ncbi.nlm.nih.gov/pubmed?term=%28%28%22antioxidants%22%5BMeSH%20Terms%5D%20AND%20%28%222011%22%5BPDAT%5D%20%3A%20%223000%22%5BPDAT%5D%29%29%20AND%20Humans%5BFilter%5D%29%20AND%20systematic%20review%5BPublication%20Type%5D&cmd=DetailsSearch) (17/09/2019) | [(((antioxidants[MeSH Terms]) AND ("2011"[Date - Publication] : "3000"[Date - Publication])) AND Humans[Filter]) AND systematic review[Publication Type]](https://www.ncbi.nlm.nih.gov/pubmed?term=%28%28%22antioxidants%22%5BMeSH%20Terms%5D%20AND%20%28%222011%22%5BPDAT%5D%20%3A%20%223000%22%5BPDAT%5D%29%29%20AND%20Humans%5BFilter%5D%29%20AND%20systematic%20review%5BPublication%20Type%5D&cmd=DetailsSearch) |
| Alcohol/ethanol | [239](https://www.ncbi.nlm.nih.gov/pubmed?term=%28%28%28alcohol%5BTitle%5D%20OR%20ethanol%5BTitle%5D%29%20AND%20%28review%5BPublication%20Type%5D%20AND%20%28%222011%22%5BPDAT%5D%20%3A%20%223000%22%5BPDAT%5D%29%29%29%20AND%20Humans%5BFilter%5D%29%20AND%20%28%28%22diet%22%5BMeSH%20Terms%5D%20OR%20%22diet%22%5BAll%20Fields%5D%29%20OR%20%28%22diet%22%5BMeSH%20Terms%5D%20OR%20%22diet%22%5BAll%20Fields%5D%20OR%20%22dietary%22%5BAll%20Fields%5D%29%20OR%20%28%22food%22%5BMeSH%20Terms%5D%20OR%20%22food%22%5BAll%20Fields%5D%29%20OR%20%28%22nutritional%20status%22%5BMeSH%20Terms%5D%20OR%20%28%22nutritional%22%5BAll%20Fields%5D%20AND%20%22status%22%5BAll%20Fields%5D%29%20OR%20%22nutritional%20status%22%5BAll%20Fields%5D%20OR%20%22nutrition%22%5BAll%20Fields%5D%20OR%20%22nutritional%20sciences%22%5BMeSH%20Terms%5D%20OR%20%28%22nutritional%22%5BAll%20Fields%5D%20AND%20%22sciences%22%5BAll%20Fields%5D%29%20OR%20%22nutritional%20sciences%22%5BAll%20Fields%5D%29%20OR%20Nutritional%5BAll%20Fields%5D%29&cmd=DetailsSearch) (17/09/2019) | [(alcohol[Title] OR ethanol[Title]) AND review[Publication Type] AND ("2011"[Date - Publication] : "3000"[Date - Publication]) AND Humans[Filter] AND (Diet OR Dietary OR Food OR Nutrition OR Nutritional)](https://www.ncbi.nlm.nih.gov/pubmed/?term=(alcohol%5BTitle%5D+OR+ethanol%5BTitle%5D)+AND+review%5BPublication+Type%5D+AND+(%222011%22%5BDate+-+Publication%5D+%3A+%223000%22%5BDate+-+Publication%5D)+AND+Humans%5BFilter%5D+AND+(Diet+OR+Dietary+OR+Food+OR+Nutrition+OR+Nutritional)) |
| Fluid/water balance | [146](https://pubmed.ncbi.nlm.nih.gov/?term=%28balance%2C+water+electrolyte%5BMeSH+Terms%5D+OR+%22water+balance%22%5BTitle%2FAbstract%5D+OR+%22fluid+balance%22%5BTitle%2FAbstract%5D+OR+%22hydration%22%5BTitle%2FAbstract%5D+OR+%22water+intake%22%5BTitle%2FAbstract%5D%29+AND+%28%222011%22%5BDate+-+Publication%5D+%3A+%223000%22%5BDate+-+Publication%5D%29+AND+Humans%5BFilter%5D+AND+Systematic+Review%5BPublication+Type%5D&filter=pubt.systematicreviews&sort=pubdate) | [(balance, water electrolyte[MeSH Terms] OR "water balance"[Title/Abstract] OR "fluid balance"[Title/Abstract] OR "hydration"[Title/Abstract] OR "water intake"[Title/Abstract]) AND ("2011"[Date - Publication] : "3000"[Date - Publication]) AND Humans[Filter](https://pubmed.ncbi.nlm.nih.gov/?term=%28balance%2C+water+electrolyte%5BMeSH+Terms%5D+OR+%22water+balance%22%5BTitle%2FAbstract%5D+OR+%22fluid+balance%22%5BTitle%2FAbstract%5D+OR+%22hydration%22%5BTitle%2FAbstract%5D+OR+%22water+intake%22%5BTitle%2FAbstract%5D%29+AND+%28%222011%22%5BDate+-+Publication%5D+%3A+%223000%22%5BDate+-+Publication%5D%29+AND+Humans%5BFilter%5D+AND+Systematic+Review%5BPublication+Type%5D&filter=pubt.systematicreviews&sort=pubdate) |
| Vitamin A | [158](https://www.ncbi.nlm.nih.gov/sites/myncbi/1t5vVRoFiKeQj/collections/58864606/public/) (17/09/2019) | [(((((("vitamin a"[Title])) AND (review[Publication Type])) AND ("2011"[Date - Publication] : "3000"[Date - Publication]))) AND Humans[Filter])](https://www.ncbi.nlm.nih.gov/pubmed?term=%28%28%22vitamin%20a%22%5BTitle%5D%20AND%20review%5BPublication%20Type%5D%29%20AND%20%28%222011%22%5BPDAT%5D%20%3A%20%223000%22%5BPDAT%5D%29%29%20AND%20Humans%5BFilter%5D&cmd=DetailsSearch) |
| Vitamin D | [232](https://www.ncbi.nlm.nih.gov/pubmed/?term=((((((((%22vitamin+d%22%5BTitle%5D))+AND+(%222011%22%5BDate+-+Publication%5D+%3A+%223000%22%5BDate+-+Publication%5D))+AND+Humans%5BFilter%5D)+AND+systematic+review%5BPublication+Type%5D)))+AND+(Diet+OR+Dietary+OR+Food+OR+Nutrition+OR+Nutritional))) (17/09/2019) | ["vitamin d"[Title] AND ("2011"[Date - Publication] : "3000"[Date - Publication]) AND Humans[Filter] AND systematic review[Publication Type] AND (Diet OR Dietary OR Food OR Nutrition OR Nutritional)](https://www.ncbi.nlm.nih.gov/pubmed/?term=%22vitamin+d%22%5BTitle%5D+AND+(%222011%22%5BDate+-+Publication%5D+%3A+%223000%22%5BDate+-+Publication%5D)+AND+Humans%5BFilter%5D+AND+systematic+review%5BPublication+Type%5D+AND+(Diet+OR+Dietary+OR+Food+OR+Nutrition+OR+Nutritional)) |
| Vitamin E | [488](https://d.docs.live.net/f6020bf5d433e810/Jobb/NNR6/(vitamin%20e%5bMeSH%20Terms%5d%20OR%20%22vitamin%20e%22%5bTitle%5d)%20AND%20(%222011%22%5bDate%20-%20Publication%5d%20:%20%223000%22%5bDate%20-%20Publication%5d)%20AND%20Humans%5bFilter%5d%20AND%20review%5bPublication%20Type%5d) (17/09/2019) | [(vitamin e[MeSH Terms] OR "vitamin e"[Title]) AND ("2011"[Date - Publication] : "3000"[Date - Publication]) AND Humans[Filter] AND review[Publication Type]](https://www.ncbi.nlm.nih.gov/pubmed/?term=(vitamin+e%5BMeSH+Terms%5D+OR+%22vitamin+e%22%5BTitle%5D)+AND+(%222011%22%5BDate+-+Publication%5D+%3A+%223000%22%5BDate+-+Publication%5D)+AND+Humans%5BFilter%5D+AND+review%5BPublication+Type%5D) |
| Vitamin K | [176](https://www.ncbi.nlm.nih.gov/pubmed?term=%28%28%22vitamin%20k%22%5BMeSH%20Terms%5D%20OR%20%22vitamin%20k%22%5BTitle%5D%29%20AND%20%28%28%28%222011%22%5BPDAT%5D%20%3A%20%223000%22%5BPDAT%5D%29%20AND%20Humans%5BFilter%5D%29%20AND%20review%5BPublication%20Type%5D%29%29%20AND%20%28%28%22diet%22%5BMeSH%20Terms%5D%20OR%20%22diet%22%5BAll%20Fields%5D%29%20OR%20%28%22diet%22%5BMeSH%20Terms%5D%20OR%20%22diet%22%5BAll%20Fields%5D%20OR%20%22dietary%22%5BAll%20Fields%5D%29%20OR%20%28%22food%22%5BMeSH%20Terms%5D%20OR%20%22food%22%5BAll%20Fields%5D%29%20OR%20%28%22nutritional%20status%22%5BMeSH%20Terms%5D%20OR%20%28%22nutritional%22%5BAll%20Fields%5D%20AND%20%22status%22%5BAll%20Fields%5D%29%20OR%20%22nutritional%20status%22%5BAll%20Fields%5D%20OR%20%22nutrition%22%5BAll%20Fields%5D%20OR%20%22nutritional%20sciences%22%5BMeSH%20Terms%5D%20OR%20%28%22nutritional%22%5BAll%20Fields%5D%20AND%20%22sciences%22%5BAll%20Fields%5D%29%20OR%20%22nutritional%20sciences%22%5BAll%20Fields%5D%29%20OR%20Nutritional%5BAll%20Fields%5D%29&cmd=DetailsSearch) (17/09/2019) | [(((("vitamin k"[MeSH Terms] OR "vitamin k"[Title]) AND ((("2011"[PDAT] : "3000"[PDAT]) AND Humans[Filter]) AND review[Publication Type])))) AND ((Diet OR Dietary OR Food OR Nutrition OR Nutritional))](https://www.ncbi.nlm.nih.gov/pubmed?term=%28%28%22vitamin%20k%22%5BMeSH%20Terms%5D%20OR%20%22vitamin%20k%22%5BTitle%5D%29%20AND%20%28%28%28%222011%22%5BPDAT%5D%20%3A%20%223000%22%5BPDAT%5D%29%20AND%20Humans%5BFilter%5D%29%20AND%20review%5BPublication%20Type%5D%29%29%20AND%20%28%28%22diet%22%5BMeSH%20Terms%5D%20OR%20%22diet%22%5BAll%20Fields%5D%29%20OR%20%28%22diet%22%5BMeSH%20Terms%5D%20OR%20%22diet%22%5BAll%20Fields%5D%20OR%20%22dietary%22%5BAll%20Fields%5D%29%20OR%20%28%22food%22%5BMeSH%20Terms%5D%20OR%20%22food%22%5BAll%20Fields%5D%29%20OR%20%28%22nutritional%20status%22%5BMeSH%20Terms%5D%20OR%20%28%22nutritional%22%5BAll%20Fields%5D%20AND%20%22status%22%5BAll%20Fields%5D%29%20OR%20%22nutritional%20status%22%5BAll%20Fields%5D%20OR%20%22nutrition%22%5BAll%20Fields%5D%20OR%20%22nutritional%20sciences%22%5BMeSH%20Terms%5D%20OR%20%28%22nutritional%22%5BAll%20Fields%5D%20AND%20%22sciences%22%5BAll%20Fields%5D%29%20OR%20%22nutritional%20sciences%22%5BAll%20Fields%5D%29%20OR%20Nutritional%5BAll%20Fields%5D%29&cmd=DetailsSearch) |
| Thiamine | [142](https://www.ncbi.nlm.nih.gov/pubmed/?term=(thiamine%5BMeSH+Terms%5D+OR+Thiamine%5BTitle%5D)+AND+review%5BPublication+Type%5D+AND+(%222011%22%5BDate+-+Publication%5D+%3A+%223000%22%5BDate+-+Publication%5D)+AND+humans%5BFilter%5D) (18/09/2019) | [(thiamine[MeSH Terms] OR Thiamine[Title]) AND review[Publication Type] AND ("2011"[Date - Publication] : "3000"[Date - Publication]) AND humans[Filter]](https://www.ncbi.nlm.nih.gov/pubmed/?term=(thiamine%5BMeSH+Terms%5D+OR+Thiamine%5BTitle%5D)+AND+review%5BPublication+Type%5D+AND+(%222011%22%5BDate+-+Publication%5D+%3A+%223000%22%5BDate+-+Publication%5D)+AND+humans%5BFilter%5D) |
| Riboflavin | [161](https://www.ncbi.nlm.nih.gov/pubmed/?term=(riboflavin%5BMeSH+Terms%5D+OR+riboflavin%5BTitle%5D)+AND+review%5BPublication+Type%5D+AND+(%222011%22%5BDate+-+Publication%5D+%3A+%223000%22%5BDate+-+Publication%5D)+AND+humans%5BFilter%5D) (18/09/2019) | [(riboflavin[MeSH Terms] OR riboflavin[Title]) AND review[Publication Type] AND ("2011"[Date - Publication] : "3000"[Date - Publication]) AND humans[Filter]](https://www.ncbi.nlm.nih.gov/pubmed/?term=(riboflavin%5BMeSH+Terms%5D+OR+riboflavin%5BTitle%5D)+AND+review%5BPublication+Type%5D+AND+(%222011%22%5BDate+-+Publication%5D+%3A+%223000%22%5BDate+-+Publication%5D)+AND+humans%5BFilter%5D) |
| Niacin | [156](https://www.ncbi.nlm.nih.gov/pubmed/?term=(Niacin%5BMeSH+Terms%5D+OR+Niacin%5BTitle%5D)+AND+review%5BPublication+Type%5D+AND+(%222011%22%5BDate+-+Publication%5D+%3A+%223000%22%5BDate+-+Publication%5D)+AND+humans%5BFilter%5D) (18/09/2019) | [(Niacin[MeSH Terms] OR Niacin[Title]) AND review[Publication Type] AND ("2011"[Date - Publication] : "3000"[Date - Publication]) AND humans[Filter]](https://www.ncbi.nlm.nih.gov/pubmed/?term=(Niacin%5BMeSH+Terms%5D+OR+Niacin%5BTitle%5D)+AND+review%5BPublication+Type%5D+AND+(%222011%22%5BDate+-+Publication%5D+%3A+%223000%22%5BDate+-+Publication%5D)+AND+humans%5BFilter%5D) |
| Vitamin B6 | [146](https://www.ncbi.nlm.nih.gov/pubmed/?term=(vitamin+b6%5BMeSH+Terms%5D)+OR+pyridoxine%5BMeSH+Terms%5D+OR+%22vitamin+b6%22%5BTitle%5D+OR+pyridoxine%5BTitle%5D)+AND+review%5BPublication+Type%5D)+AND+(%222011%22%5BDate+-+Publication%5D+%3A+%223000%22%5BDate+-+Publication%5D)+AND+humans%5BFilter%5D) (18/09/2019) | [(vitamin b6[MeSH Terms]) OR pyridoxine[MeSH Terms] OR "vitamin b6"[Title] OR pyridoxine[Title]) AND review[Publication Type]) AND ("2011"[Date - Publication] : "3000"[Date - Publication]) AND humans[Filter]](https://www.ncbi.nlm.nih.gov/pubmed/?term=(vitamin+b6%5BMeSH+Terms%5D)+OR+pyridoxine%5BMeSH+Terms%5D+OR+%22vitamin+b6%22%5BTitle%5D+OR+pyridoxine%5BTitle%5D)+AND+review%5BPublication+Type%5D)+AND+(%222011%22%5BDate+-+Publication%5D+%3A+%223000%22%5BDate+-+Publication%5D)+AND+humans%5BFilter%5D) |
| Folate | [466](https://www.ncbi.nlm.nih.gov/pubmed?term=((folate%5BMeSH+Terms%5D+AND+review%5BPublication+Type%5D+AND+(%222011%22%5BDate+-+Publication%5D+%3A+%223000%22%5BDate+-+Publication%5D)+AND+Humans%5BFilter%5D))+AND+((%22Diet%22+OR+%22Dietary%22+OR+%22Food%22+OR+%22Nutrition%22+OR+%22Nutritional%22))&cmd=DetailsSearch) (18/09/2019) | [((folate[MeSH Terms] AND review[Publication Type] AND ("2011"[Date - Publication] : "3000"[Date - Publication]) AND Humans[Filter])) AND (("Diet" OR "Dietary" OR "Food" OR "Nutrition" OR "Nutritional"))](https://www.ncbi.nlm.nih.gov/pubmed?term=((folate%5BMeSH+Terms%5D+AND+review%5BPublication+Type%5D+AND+(%222011%22%5BDate+-+Publication%5D+%3A+%223000%22%5BDate+-+Publication%5D)+AND+Humans%5BFilter%5D))+AND+((%22Diet%22+OR+%22Dietary%22+OR+%22Food%22+OR+%22Nutrition%22+OR+%22Nutritional%22))&cmd=DetailsSearch) |
| Vitamin B12 | [337](https://www.ncbi.nlm.nih.gov/pubmed/?term=(b12%2C+vitamin%5BMeSH+Terms%5D+OR+cobalamin%5BMeSH+Terms%5D)+AND+review%5BPublication+Type%5D+AND+(%222011%22%5BDate+-+Publication%5D+%3A+%223000%22%5BDate+-+Publication%5D)+AND+Humans%5BFilter%5D) (18/09/2019) | [(b12, vitamin[MeSH Terms] OR cobalamin[MeSH Terms]) AND review[Publication Type] AND ("2011"[Date - Publication] : "3000"[Date - Publication]) AND Humans[Filter]](https://www.ncbi.nlm.nih.gov/pubmed/?term=(b12%2C+vitamin%5BMeSH+Terms%5D+OR+cobalamin%5BMeSH+Terms%5D)+AND+review%5BPublication+Type%5D+AND+(%222011%22%5BDate+-+Publication%5D+%3A+%223000%22%5BDate+-+Publication%5D)+AND+Humans%5BFilter%5D) |
| Biotin | [168](https://www.ncbi.nlm.nih.gov/pubmed?term=%28%22biotin%22%5BMeSH%20Terms%5D%20OR%20%22biotin%22%5BAll%20Fields%5D%29%20AND%20review%5BPublication%20Type%5D%20AND%20%28%222011%22%5BPDAT%5D%20%3A%20%223000%22%5BPDAT%5D%29%20AND%20Humans%5BFilter%5D&cmd=DetailsSearch) (18/09/2019) | [("biotin"[MeSH Terms] OR "biotin"[All Fields]) AND review[Publication Type] AND ("2011"[PDAT] : "3000"[PDAT]) AND Humans[Filter]](https://www.ncbi.nlm.nih.gov/pubmed?term=%28%22biotin%22%5BMeSH%20Terms%5D%20OR%20%22biotin%22%5BAll%20Fields%5D%29%20AND%20review%5BPublication%20Type%5D%20AND%20%28%222011%22%5BPDAT%5D%20%3A%20%223000%22%5BPDAT%5D%29%20AND%20Humans%5BFilter%5D&cmd=DetailsSearch) |
| Pantothenic acid | [16](https://www.ncbi.nlm.nih.gov/pubmed/?term=(pantothenic+acid%5BMeSH+Terms%5D+AND+(%222011%22%5BDate+-+Publication%5D+%3A+%223000%22%5BDate+-+Publication%5D)+AND+review%5BPublication+Type%5D)+AND+Humans%5BFilter%5D) (19/09/2019) | [("pantothenic acid"[MeSH Terms] AND ("2011"[PDAT] : "3000"[PDAT]) AND review[Publication Type]) AND Humans[Filter]](https://www.ncbi.nlm.nih.gov/pubmed/?term=(pantothenic+acid%5BMeSH+Terms%5D+AND+(%222011%22%5BDate+-+Publication%5D+%3A+%223000%22%5BDate+-+Publication%5D)+AND+review%5BPublication+Type%5D)+AND+Humans%5BFilter%5D) |
| Vitamin C | [420](https://www.ncbi.nlm.nih.gov/pubmed/?term=((vitamin+c%5BMeSH+Terms%5D+OR+ascorbic+acid%5BMeSH+Terms%5D+OR+dehydroascorbic+acid%5BMeSH+Terms%5D)+AND+(%222011%22%5BDate+-+Publication%5D+%3A+%223000%22%5BDate+-+Publication%5D)+AND+review%5BPublication+Type%5D+AND+Humans%5BFilter%5D)) (19/09/2019) | [((vitamin c[MeSH Terms] OR ascorbic acid[MeSH Terms] OR dehydroascorbic acid[MeSH Terms]) AND ("2011"[Date - Publication] : "3000"[Date - Publication]) AND review[Publication Type] AND Humans[Filter])](https://www.ncbi.nlm.nih.gov/pubmed/?term=((vitamin+c%5BMeSH+Terms%5D+OR+ascorbic+acid%5BMeSH+Terms%5D+OR+dehydroascorbic+acid%5BMeSH+Terms%5D)+AND+(%222011%22%5BDate+-+Publication%5D+%3A+%223000%22%5BDate+-+Publication%5D)+AND+review%5BPublication+Type%5D+AND+Humans%5BFilter%5D)) |
| Choline | [413](https://pubmed.ncbi.nlm.nih.gov/?term=%22choline%22%5BMeSH+Terms%5D+AND+%28%222011%22%5BDate+-+Publication%5D+%3A+%223000%22%5BDate+-+Publication%5D%29+AND+Humans%5BFilter%5D+AND+Review%5BPublication+Type%5D&sort=pubdate&size=50) (17/02/2020) | ["choline"[MeSH Terms] AND ("2011"[Date - Publication] : "3000"[Date - Publication]) AND Humans[Filter] AND Review[Publication Type]](https://pubmed.ncbi.nlm.nih.gov/?term=%22choline%22%5BMeSH+Terms%5D+AND+%28%222011%22%5BDate+-+Publication%5D+%3A+%223000%22%5BDate+-+Publication%5D%29+AND+Humans%5BFilter%5D+AND+Review%5BPublication+Type%5D&sort=pubdate&size=50) |
| Calcium | [385](https://www.ncbi.nlm.nih.gov/pubmed/?term=calcium%2C+dietary%5BMeSH+Terms%5D+AND+(%222011%22%5BDate+-+Publication%5D+%3A+%223000%22%5BDate+-+Publication%5D)+AND+review%5BPublication+Type%5D+AND+Humans%5BFilter%5D) (19/09/2019) | [(("calcium, dietary"[MeSH Terms] AND ("2011"[PDAT] : "3000"[PDAT])) AND review[Publication Type]) AND Humans[Filter]](https://www.ncbi.nlm.nih.gov/pubmed/?term=calcium%2C+dietary%5BMeSH+Terms%5D+AND+(%222011%22%5BDate+-+Publication%5D+%3A+%223000%22%5BDate+-+Publication%5D)+AND+review%5BPublication+Type%5D+AND+Humans%5BFilter%5D) |
| Phosphorus | [308](https://www.ncbi.nlm.nih.gov/pubmed/?term=(%22phosphorus%2C%20dietary%22%5BMeSH%20Terms%5D%20OR%20%22phosphorus%22%5BMeSH%20Terms%5D)%20AND%20(%222011%22%5BPDAT%5D%20%3A%20%223000%22%5BPDAT%5D)%20AND%20review%5BPublication%20Type%5D%20AND%20Humans%5BFilter%5D&cmd=DetailsSearch) (19/09/2019)  (With [“phosphorus, dietary[MeSH Terms] only](https://www.ncbi.nlm.nih.gov/pubmed/?term=%22phosphorus%2C%20dietary%22%5BMeSH%20Terms%5D%20AND%20(%222011%22%5BPDAT%5D%20%3A%20%223000%22%5BPDAT%5D)%20AND%20review%5BPublication%20Type%5D%20AND%20Humans%5BFilter%5D&cmd=DetailsSearch): [89](https://www.ncbi.nlm.nih.gov/pubmed/?term=%22phosphorus%2C%20dietary%22%5BMeSH%20Terms%5D%20AND%20(%222011%22%5BPDAT%5D%20%3A%20%223000%22%5BPDAT%5D)%20AND%20review%5BPublication%20Type%5D%20AND%20Humans%5BFilter%5D&cmd=DetailsSearch)) | [("phosphorus, dietary"[MeSH Terms] OR "phosphorus"[MeSH Terms]) AND ("2011"[PDAT] : "3000"[PDAT]) AND review[Publication Type] AND Humans[Filter](https://www.ncbi.nlm.nih.gov/pubmed/?term=(%22phosphorus%2C%20dietary%22%5BMeSH%20Terms%5D%20OR%20%22phosphorus%22%5BMeSH%20Terms%5D)%20AND%20(%222011%22%5BPDAT%5D%20%3A%20%223000%22%5BPDAT%5D)%20AND%20review%5BPublication%20Type%5D%20AND%20Humans%5BFilter%5D&cmd=DetailsSearch)] |
| Magnesium | 470 (19/09/2019) | [(magnesium[MeSH Terms] AND ("2011"[Date - Publication] : "3000"[Date - Publication]) AND review[Publication Type] AND Humans[Filter])](https://www.ncbi.nlm.nih.gov/pubmed?term=(magnesium%5BMeSH%20Terms%5D%20AND%20(%222011%22%5BDate%20-%20Publication%5D%20%3A%20%223000%22%5BDate%20-%20Publication%5D)%20AND%20review%5BPublication%20Type%5D%20AND%20Humans%5BFilter%5D)) |
| Sodium as salt | [210](https://www.ncbi.nlm.nih.gov/pubmed/?term=sodium%5BTitle%5D+AND+(%222011%22%5BDate+-+Publication%5D+%3A+%223000%22%5BDate+-+Publication%5D)+AND+review%5BPublication+Type%5D+AND+Humans%5BFilter%5D+AND+(%22Diet%22+OR+%22Dietary%22+OR+%22Food%22+OR+%22Nutrition%22+OR+%22Nutritional%22)) (19/09/2019) | [sodium[Title] AND ("2011"[Date - Publication] : "3000"[Date - Publication]) AND review[Publication Type] AND Humans[Filter] AND ("Diet" OR "Dietary" OR "Food" OR "Nutrition" OR "Nutritional")](https://www.ncbi.nlm.nih.gov/pubmed/?term=sodium%5BTitle%5D+AND+(%222011%22%5BDate+-+Publication%5D+%3A+%223000%22%5BDate+-+Publication%5D)+AND+review%5BPublication+Type%5D+AND+Humans%5BFilter%5D+AND+(%22Diet%22+OR+%22Dietary%22+OR+%22Food%22+OR+%22Nutrition%22+OR+%22Nutritional%22)) |
| Potassium | [496](https://www.ncbi.nlm.nih.gov/pubmed/?term=(%22potassium%22%5BMeSH+Terms%5D+OR+dietary+potassium%5BMeSH+Terms%5D)+AND+(%222011%22%5BDate+-+Publication%5D+%3A+%223000%22%5BDate+-+Publication%5D)+AND+review%5BPublication+Type%5D+AND+Humans%5BFilter%5D) (19/09/2019)  (With “dietary potassium[MeSH Terms] only = [73](https://www.ncbi.nlm.nih.gov/pubmed/?term=dietary+potassium%5BMeSH+Terms%5D+AND+(%222011%22%5BDate+-+Publication%5D+%3A+%223000%22%5BDate+-+Publication%5D)+AND+review%5BPublication+Type%5D+AND+Humans%5BFilter%5D)) | [("potassium"[MeSH Terms] OR dietary potassium[MeSH Terms]) AND ("2011"[Date - Publication] : "3000"[Date - Publication]) AND review[Publication Type] AND Humans[Filter]](https://www.ncbi.nlm.nih.gov/pubmed/?term=(%22potassium%22%5BMeSH+Terms%5D+OR+dietary+potassium%5BMeSH+Terms%5D)+AND+(%222011%22%5BDate+-+Publication%5D+%3A+%223000%22%5BDate+-+Publication%5D)+AND+review%5BPublication+Type%5D+AND+Humans%5BFilter%5D) |
| Iron | [479](https://www.ncbi.nlm.nih.gov/pubmed/?term=iron%5BMeSH+Terms%5D+AND+(%222011%22%5BDate+-+Publication%5D+%3A+%223000%22%5BDate+-+Publication%5D)+AND+review%5BPublication+Type%5D+AND+Humans%5BFilter%5D+AND+(%22Diet%22+OR+%22Dietary%22+OR+%22Food%22+OR+%22Nutrition%22+OR+%22Nutritional%22)) (19/09/2019 | [iron[MeSH Terms] AND ("2011"[Date - Publication] : "3000"[Date - Publication]) AND review[Publication Type] AND Humans[Filter] AND ("Diet" OR "Dietary" OR "Food" OR "Nutrition" OR "Nutritional")](https://www.ncbi.nlm.nih.gov/pubmed/?term=iron%5BMeSH+Terms%5D+AND+(%222011%22%5BDate+-+Publication%5D+%3A+%223000%22%5BDate+-+Publication%5D)+AND+review%5BPublication+Type%5D+AND+Humans%5BFilter%5D+AND+(%22Diet%22+OR+%22Dietary%22+OR+%22Food%22+OR+%22Nutrition%22+OR+%22Nutritional%22)) |
| Zinc | [381](https://www.ncbi.nlm.nih.gov/pubmed/?term=zinc%5BMeSH+Terms%5D+AND+(%222011%22%5BDate+-+Publication%5D+%3A+%223000%22%5BDate+-+Publication%5D)+AND+review%5BPublication+Type%5D+AND+Humans%5BFilter%5D+AND+(%22Diet%22+OR+%22Dietary%22+OR+%22Food%22+OR+%22Nutrition%22+OR+%22Nutritional%22)) (19/09/2019) | [zinc[MeSH Terms] AND ("2011"[Date - Publication] : "3000"[Date - Publication]) AND review[Publication Type] AND Humans[Filter] AND ("Diet" OR "Dietary" OR "Food" OR "Nutrition" OR "Nutritional")](https://www.ncbi.nlm.nih.gov/pubmed/?term=zinc%5BMeSH+Terms%5D+AND+(%222011%22%5BDate+-+Publication%5D+%3A+%223000%22%5BDate+-+Publication%5D)+AND+review%5BPublication+Type%5D+AND+Humans%5BFilter%5D+AND+(%22Diet%22+OR+%22Dietary%22+OR+%22Food%22+OR+%22Nutrition%22+OR+%22Nutritional%22)) |
| Iodine | [188](https://www.ncbi.nlm.nih.gov/pubmed/?term=iodine%5BMeSH+Terms%5D+AND+(%222011%22%5BDate+-+Publication%5D+%3A+%223000%22%5BDate+-+Publication%5D)+AND+review%5BPublication+Type%5D+AND+Humans%5BFilter%5D+AND+(%22Diet%22+OR+%22Dietary%22+OR+%22Food%22+OR+%22Nutrition%22+OR+%22Nutritional%22)) (19/09/2019) | [iodine[MeSH Terms] AND ("2011"[Date - Publication] : "3000"[Date - Publication]) AND review[Publication Type] AND Humans[Filter] AND ("Diet" OR "Dietary" OR "Food" OR "Nutrition" OR "Nutritional")](https://www.ncbi.nlm.nih.gov/pubmed/?term=iodine%5BMeSH+Terms%5D+AND+(%222011%22%5BDate+-+Publication%5D+%3A+%223000%22%5BDate+-+Publication%5D)+AND+review%5BPublication+Type%5D+AND+Humans%5BFilter%5D+AND+(%22Diet%22+OR+%22Dietary%22+OR+%22Food%22+OR+%22Nutrition%22+OR+%22Nutritional%22)) |
| Selenium | [393](https://www.ncbi.nlm.nih.gov/pubmed/?term=%22selenium%22%5BMeSH%20Terms%5D%20AND%20(%222011%22%5BPDAT%5D%20%3A%20%223000%22%5BPDAT%5D)%20AND%20review%5BPublication%20Type%5D%20AND%20Humans%5BFilter%5D&cmd=DetailsSearch) (19/09/2019) | ["selenium"[MeSH Terms] AND ("2011"[PDAT] : "3000"[PDAT]) AND review[Publication Type] AND Humans[Filter]](https://www.ncbi.nlm.nih.gov/pubmed/?term=%22selenium%22%5BMeSH%20Terms%5D%20AND%20(%222011%22%5BPDAT%5D%20%3A%20%223000%22%5BPDAT%5D)%20AND%20review%5BPublication%20Type%5D%20AND%20Humans%5BFilter%5D&cmd=DetailsSearch) |
| Copper | [484](https://www.ncbi.nlm.nih.gov/pubmed/?term=copper%5BMeSH+Terms%5D+AND+(%222011%22%5BDate+-+Publication%5D+%3A+%223000%22%5BDate+-+Publication%5D)+AND+review%5BPublication+Type%5D+AND+Humans%5BFilter%5D) (19/09/2019) | [copper[MeSH Terms] AND ("2011"[Date - Publication] : "3000"[Date - Publication]) AND review[Publication Type] AND Humans[Filter]](https://www.ncbi.nlm.nih.gov/pubmed/?term=copper%5BMeSH+Terms%5D+AND+(%222011%22%5BDate+-+Publication%5D+%3A+%223000%22%5BDate+-+Publication%5D)+AND+review%5BPublication+Type%5D+AND+Humans%5BFilter%5D) |
| Chromium | [122](https://www.ncbi.nlm.nih.gov/pubmed/?term=chromium%5BMeSH+Terms%5D+AND+(%222011%22%5BDate+-+Publication%5D+%3A+%223000%22%5BDate+-+Publication%5D)+AND+review%5BPublication+Type%5D+AND+Humans%5BFilter%5D) (19/09/2019) | [chromium[MeSH Terms] AND ("2011"[Date - Publication] : "3000"[Date - Publication]) AND review[Publication Type] AND Humans[Filter]](https://www.ncbi.nlm.nih.gov/pubmed/?term=chromium%5BMeSH+Terms%5D+AND+(%222011%22%5BDate+-+Publication%5D+%3A+%223000%22%5BDate+-+Publication%5D)+AND+review%5BPublication+Type%5D+AND+Humans%5BFilter%5D) |
| Manganese | [157](https://www.ncbi.nlm.nih.gov/pubmed/?term=%22manganese%22%5BMeSH%20Terms%5D%20AND%20(%222011%22%5BPDAT%5D%20%3A%20%223000%22%5BPDAT%5D)%20AND%20review%5BPublication%20Type%5D%20AND%20Humans%5BFilter%5D&cmd=DetailsSearch) (19/09/2019) | ["manganese"[MeSH Terms] AND ("2011"[PDAT] : "3000"[PDAT]) AND review[Publication Type] AND Humans[Filter]](https://www.ncbi.nlm.nih.gov/pubmed/?term=%22manganese%22%5BMeSH%20Terms%5D%20AND%20(%222011%22%5BPDAT%5D%20%3A%20%223000%22%5BPDAT%5D)%20AND%20review%5BPublication%20Type%5D%20AND%20Humans%5BFilter%5D&cmd=DetailsSearch) |
| Molybdenum | [44](https://www.ncbi.nlm.nih.gov/pubmed/?term=%22molybdenum%22%5BMeSH+Terms%5D+AND+(%222011%22%5BPDAT%5D+%3A+%223000%22%5BPDAT%5D)+AND+review%5BPublication+Type%5D+AND+Humans%5BFilter%5D) (19/09/2019) | ["molybdenum"[MeSH Terms] AND ("2011"[PDAT] : "3000"[PDAT]) AND review[Publication Type] AND Humans[Filter]](https://www.ncbi.nlm.nih.gov/pubmed/?term=%22molybdenum%22%5BMeSH+Terms%5D+AND+(%222011%22%5BPDAT%5D+%3A+%223000%22%5BPDAT%5D)+AND+review%5BPublication+Type%5D+AND+Humans%5BFilter%5D) |
| Fluoride | [326](https://www.ncbi.nlm.nih.gov/pubmed/?term=fluoride%5BMeSH+Terms%5D+AND+(%222011%22%5BPDAT%5D+%3A+%223000%22%5BPDAT%5D)+AND+review%5BPublication+Type%5D+AND+Humans%5BFilter%5D) (19/09/2019) | [fluoride[MeSH Terms] AND ("2011"[PDAT] : "3000"[PDAT]) AND review[Publication Type] AND Humans[Filter]](https://www.ncbi.nlm.nih.gov/pubmed/?term=fluoride%5BMeSH+Terms%5D+AND+(%222011%22%5BPDAT%5D+%3A+%223000%22%5BPDAT%5D)+AND+review%5BPublication+Type%5D+AND+Humans%5BFilter%5D) |

**Foods**

| Topic | No. items | Query with link |
| --- | --- | --- |
| Breast feeding | [609](https://www.ncbi.nlm.nih.gov/pubmed/?term=(%22breast+feeding%22%5BMeSH+Terms%5D+OR+%22breast+feeding%22%5BTitle%2FAbstract%5D+OR+%22breastfeeding%22%5BTitle%2FAbstract%5D+OR+%22milk%2C+human%22%5BMeSH+Terms%5D+OR+%22breast+milk%22%5BTitle%2FAbstract%5D)+AND+(%222011%22%5BPDAT%5D+%3A+%223000%22%5BPDAT%5D)+AND+Humans%5BFilter%5D+AND+Systematic+Review%5BPublication+Type%5D+AND+(Danish%5Blang%5D+OR+English%5Blang%5D+OR+Finnish%5Blang%5D+OR+Norwegian%5Blang%5D+OR+Swedish%5Blang%5D))  (16/10/2019) | [("breast feeding"[MeSH Terms] OR "breast feeding"[Title/Abstract] OR "breastfeeding"[Title/Abstract] OR "milk, human"[MeSH Terms] OR "breast milk"[Title/Abstract]) AND ("2011"[PDAT] : "3000"[PDAT]) AND Humans[Filter] AND Systematic Review[Publication Type] AND (Danish[lang] OR English[lang] OR Finnish[lang] OR Norwegian[lang] OR Swedish[lang])](https://www.ncbi.nlm.nih.gov/pubmed/?term=(%22breast+feeding%22%5BMeSH+Terms%5D+OR+%22breast+feeding%22%5BTitle%2FAbstract%5D+OR+%22breastfeeding%22%5BTitle%2FAbstract%5D+OR+%22milk%2C+human%22%5BMeSH+Terms%5D+OR+%22breast+milk%22%5BTitle%2FAbstract%5D)+AND+(%222011%22%5BPDAT%5D+%3A+%223000%22%5BPDAT%5D)+AND+Humans%5BFilter%5D+AND+Systematic+Review%5BPublication+Type%5D+AND+(Danish%5Blang%5D+OR+English%5Blang%5D+OR+Finnish%5Blang%5D+OR+Norwegian%5Blang%5D+OR+Swedish%5Blang%5D)) |
| Cereals | [169](https://www.ncbi.nlm.nih.gov/pubmed/?term=(cereal*%5BTitle%5D+OR+grain*%5BTitle%5D+OR+%22whole+grain*%22%5BTitle%5D)+AND+(%222011%22%5BDate+-+Publication%5D+%3A+%223000%22%5BDate+-+Publication%5D)+AND+humans%5BFilter%5D+AND+review%5BPublication+Type%5D) (26/09/2019) | [(cereal*[Title] OR grain*[Title] OR "whole grain*"[Title]) AND ("2011"[Date - Publication] : "3000"[Date - Publication]) AND humans[Filter] AND review[Publication Type]](https://www.ncbi.nlm.nih.gov/pubmed/?term=(cereal*%5BTitle%5D+OR+grain*%5BTitle%5D+OR+%22whole+grain*%22%5BTitle%5D)+AND+(%222011%22%5BDate+-+Publication%5D+%3A+%223000%22%5BDate+-+Publication%5D)+AND+humans%5BFilter%5D+AND+review%5BPublication+Type%5D) |
| Vegetables, fruits, berries | [449](https://www.ncbi.nlm.nih.gov/pubmed/?term=(fruit*%5BTitle%5D+OR+vegetable*%5BTitle%5D+OR+berry%5BTitle%5D+OR+berries%5BTitle%5D+OR+spice*%5BTitle%5D+OR+potato*%5BTitle%5D)+AND+(%222011%22%5BDate+-+Publication%5D+%3A+%223000%22%5BDate+-+Publication%5D)+AND+humans%5BFilter%5D+AND+review%5BPublication+Type%5D+AND+(Diet+OR+Dietary+OR+Food+OR+Nutrition+OR+Nutritional)) (26/09/2019) | [(fruit*[Title] OR vegetable*[Title] OR berry[Title] OR berries[Title] OR spice*[Title] OR potato*[Title]) AND ("2011"[Date - Publication] : "3000"[Date - Publication]) AND humans[Filter] AND review[Publication Type] AND (Diet OR Dietary OR Food OR Nutrition OR Nutritional)](https://www.ncbi.nlm.nih.gov/pubmed/?term=(fruit*%5BTitle%5D+OR+vegetable*%5BTitle%5D+OR+berry%5BTitle%5D+OR+berries%5BTitle%5D+OR+spice*%5BTitle%5D+OR+potato*%5BTitle%5D)+AND+(%222011%22%5BDate+-+Publication%5D+%3A+%223000%22%5BDate+-+Publication%5D)+AND+humans%5BFilter%5D+AND+review%5BPublication+Type%5D+AND+(Diet+OR+Dietary+OR+Food+OR+Nutrition+OR+Nutritional)) |
| Nuts | [264](https://www.ncbi.nlm.nih.gov/pubmed/?term=(nut%5BTitle%5D+OR+nuts%5BTitle%5D+OR+%22tree+nut%22%5BTitle%5D+OR+%22tree+nuts%22%5BTitle%5D+OR+almond*%5BTitle%5D+OR+%22Brazil+nut%22%5BTitle%5D+OR+%22Brazil+nuts%22%5BTitle%5D+OR+cashew%5BTitle%5D+OR+hazelnut*%5BTitle%5D+OR+macadamia*%5BTitle%5D+OR+peanut*%5BTitle%5D+OR+pistachio*%5BTitle%5D+OR+walnut*%5BTitle%5D+OR+seeds%5BTitle%5D)+AND+(%222011%22%5BDate+-+Publication%5D+%3A+%223000%22%5BDate+-+Publication%5D)+AND+humans%5BFilter%5D+AND+review%5BPublication+Type%5D) (26/09/2019) | [(nut[Title] OR nuts[Title] OR "tree nut"[Title] OR "tree nuts"[Title] OR almond*[Title] OR "Brazil nut"[Title] OR "Brazil nuts"[Title] OR cashew[Title] OR hazelnut*[Title] OR macadamia*[Title] OR peanut*[Title] OR pistachio*[Title] OR walnut*[Title] OR seeds[Title]) AND ("2011"[Date - Publication] : "3000"[Date - Publication]) AND humans[Filter] AND review[Publication Type]](https://www.ncbi.nlm.nih.gov/pubmed/?term=(nut%5BTitle%5D+OR+nuts%5BTitle%5D+OR+%22tree+nut%22%5BTitle%5D+OR+%22tree+nuts%22%5BTitle%5D+OR+almond*%5BTitle%5D+OR+%22Brazil+nut%22%5BTitle%5D+OR+%22Brazil+nuts%22%5BTitle%5D+OR+cashew%5BTitle%5D+OR+hazelnut*%5BTitle%5D+OR+macadamia*%5BTitle%5D+OR+peanut*%5BTitle%5D+OR+pistachio*%5BTitle%5D+OR+walnut*%5BTitle%5D+OR+seeds%5BTitle%5D)+AND+(%222011%22%5BDate+-+Publication%5D+%3A+%223000%22%5BDate+-+Publication%5D)+AND+humans%5BFilter%5D+AND+review%5BPublication+Type%5D) |
| Pulses | 350 (26/09/2019) | [(pulses[MeSH Terms] OR legumes[MeSH Terms]) AND ("2011"[Date - Publication] : "3000"[Date - Publication]) AND humans[Filter] AND review[Publication Type] AND (Diet OR Dietary OR Food OR Nutrition OR Nutritional)](https://www.ncbi.nlm.nih.gov/pubmed/?term=(pulses%5BMeSH+Terms%5D+OR+legumes%5BMeSH+Terms%5D)+AND+(%222011%22%5BDate+-+Publication%5D+%3A+%223000%22%5BDate+-+Publication%5D)+AND+humans%5BFilter%5D+AND+review%5BPublication+Type%5D+AND+(Diet+OR+Dietary+OR+Food+OR+Nutrition+OR+Nutritional)) |
| Meat and meat products | [129](https://www.ncbi.nlm.nih.gov/pubmed/?term=(meat%5BMeSH+Terms%5D+OR+meats%5BMeSH+Terms%5D)+AND+(%222011%22%5BDate+-+Publication%5D+%3A+%223000%22%5BDate+-+Publication%5D)+AND+humans%5BFilter%5D+AND+systematic+review%5BPublication+Type%5D) (27/09/2019) | [(meat[MeSH Terms] OR meats[MeSH Terms]) AND ("2011"[Date - Publication] : "3000"[Date - Publication]) AND humans[Filter] AND systematic review[Publication Type]](https://www.ncbi.nlm.nih.gov/pubmed/?term=(meat%5BMeSH+Terms%5D+OR+meats%5BMeSH+Terms%5D)+AND+(%222011%22%5BDate+-+Publication%5D+%3A+%223000%22%5BDate+-+Publication%5D)+AND+humans%5BFilter%5D+AND+systematic+review%5BPublication+Type%5D)  (PS: The MeSH for “meat” includes seafood!) |
| Milk and dairy | [225](https://www.ncbi.nlm.nih.gov/pubmed/?term=(milk%5BMeSH+Terms%5D+OR+dairy+products%5BMeSH+Terms%5D)+AND+(%222011%22%5BDate+-+Publication%5D+%3A+%223000%22%5BDate+-+Publication%5D)+AND+humans%5BFilter%5D+AND+systematic+review%5BPublication+Type%5D) (27/09/2019) | [(milk[MeSH Terms] OR dairy products[MeSH Terms]) AND ("2011"[Date - Publication] : "3000"[Date - Publication]) AND humans[Filter] AND systematic review[Publication Type]](https://www.ncbi.nlm.nih.gov/pubmed/?term=(milk%5BMeSH+Terms%5D+OR+dairy+products%5BMeSH+Terms%5D)+AND+(%222011%22%5BDate+-+Publication%5D+%3A+%223000%22%5BDate+-+Publication%5D)+AND+humans%5BFilter%5D+AND+systematic+review%5BPublication+Type%5D) |
| Eggs | [103](https://www.ncbi.nlm.nih.gov/pubmed/?term=eggs%5BMeSH+Terms%5D+AND+(%222011%22%5BDate+-+Publication%5D+%3A+%223000%22%5BDate+-+Publication%5D)+AND+humans%5BFilter%5D+AND+review%5BPublication+Type%5D) (27/09/2019) | [eggs[MeSH Terms] AND ("2011"[Date - Publication] : "3000"[Date - Publication]) AND humans[Filter] AND review[Publication Type]](https://www.ncbi.nlm.nih.gov/pubmed/?term=eggs%5BMeSH+Terms%5D+AND+(%222011%22%5BDate+-+Publication%5D+%3A+%223000%22%5BDate+-+Publication%5D)+AND+humans%5BFilter%5D+AND+review%5BPublication+Type%5D) |
| Fats and oils | [154](https://www.ncbi.nlm.nih.gov/pubmed/?term=(%22dietary+fat%22%5BTitle%5D+OR+%22dietary+fats%22%5BTitle%5D+OR+butter%5BTitle%5D+OR+ghee%5BTitle%5D+OR+%22corn+oil%22%5BTitle%5D+OR+%22cottonseed+oil%22%5BTitle%5D+OR+%22canola%22%5BTitle%5D+OR+%22olive+oil%22%5BTitle%5D+OR+%22rapeseed+oil%22%5BTitle%5D+OR+%22safflower+oil%22%5BTitle%5D+OR+%22sunflower+oil%22%5BTitle%5D+OR+%22sesame+oil%22%5BTitle%5D+OR+%22soybean+oil%22%5BTitle%5D+OR+%22plant+oil%22%5BTitle%5D+OR+%22seed+oil%22%5BTitle%5D+OR+%22cooking+oil%22%5BTitle%5D+OR+%22margarine%22%5BTitle%5D)+AND+(%222011%22%5BDate+-+Publication%5D+%3A+%223000%22%5BDate+-+Publication%5D)+AND+humans%5BFilter%5D+AND+review%5BPublication+Type%5D) (27/09/2019) | [("dietary fat"[Title] OR "dietary fats"[Title] OR butter[Title] OR ghee[Title] OR "corn oil"[Title] OR "cottonseed oil"[Title] OR "canola"[Title] OR "olive oil"[Title] OR "rapeseed oil"[Title] OR "safflower oil"[Title] OR "sunflower oil"[Title] OR "sesame oil"[Title] OR "soybean oil"[Title] OR "plant oil"[Title] OR "seed oil"[Title] OR "cooking oil"[Title] OR "margarine"[Title]) AND ("2011"[Date - Publication] : "3000"[Date - Publication]) AND humans[Filter] AND review[Publication Type]](https://www.ncbi.nlm.nih.gov/pubmed/?term=(%22dietary+fat%22%5BTitle%5D+OR+%22dietary+fats%22%5BTitle%5D+OR+butter%5BTitle%5D+OR+ghee%5BTitle%5D+OR+%22corn+oil%22%5BTitle%5D+OR+%22cottonseed+oil%22%5BTitle%5D+OR+%22canola%22%5BTitle%5D+OR+%22olive+oil%22%5BTitle%5D+OR+%22rapeseed+oil%22%5BTitle%5D+OR+%22safflower+oil%22%5BTitle%5D+OR+%22sunflower+oil%22%5BTitle%5D+OR+%22sesame+oil%22%5BTitle%5D+OR+%22soybean+oil%22%5BTitle%5D+OR+%22plant+oil%22%5BTitle%5D+OR+%22seed+oil%22%5BTitle%5D+OR+%22cooking+oil%22%5BTitle%5D+OR+%22margarine%22%5BTitle%5D)+AND+(%222011%22%5BDate+-+Publication%5D+%3A+%223000%22%5BDate+-+Publication%5D)+AND+humans%5BFilter%5D+AND+review%5BPublication+Type%5D) |
| Sugar-sweetened beverages and sugar-containing foods | 124 (27/09/2019) | [("dietary sugar*"[Title] OR "sucrose"[Title] OR "candy"[Title] OR "chocolate"[Title] OR "dessert"[Title] OR "sweeten*"[Title] OR "sugar-sweetened beverage*"[Title] OR "carbonated beverage*"[Title] OR "soft drink*"[Title] OR "energy drink*"[Title] OR "fruit juice"[Title]) AND ("2011"[Date - Publication] : "3000"[Date - Publication]) AND humans[Filter] AND review[Publication Type]](https://www.ncbi.nlm.nih.gov/pubmed/?term=(%22dietary+sugar*%22%5BTitle%5D+OR+%22sucrose%22%5BTitle%5D+OR+%22candy%22%5BTitle%5D+OR+%22chocolate%22%5BTitle%5D+OR+%22dessert%22%5BTitle%5D+OR+%22sweeten*%22%5BTitle%5D+OR+%22sugar-sweetened+beverage*%22%5BTitle%5D+OR+%22carbonated+beverage*%22%5BTitle%5D+OR+%22soft+drink*%22%5BTitle%5D+OR+%22energy+drink*%22%5BTitle%5D+OR+%22fruit+juice%22%5BTitle%5D)+AND+(%222011%22%5BDate+-+Publication%5D+%3A+%223000%22%5BDate+-+Publication%5D)+AND+humans%5BFilter%5D+AND+review%5BPublication+Type%5D) |
| Sweets and confectioneries | [443](https://pubmed.ncbi.nlm.nih.gov/?term=%28dietary+sugars%5BTitle%2FAbstract%5D+OR+candy%5BTitle%2FAbstract%5D+OR+chocolate%5BTitle%2FAbstract%5D+OR+cacao%5BTitle%2FAbstract%5D+OR+sweeteners%5BTitle%2FAbstract%5D+OR+confection%2A%5BTitle%2FAbstract%5D%29+AND+%28%28%222011%22%5BDate+-+Publication%5D+%3A+%223000%22%5BDate+-+Publication%5D%29%29%29+AND+%28Humans%5BFilter%5D%29%29+AND+%28Review%5BPublication+Type%5D%29&sort=date&ac=no&fs=no) (17/02/2020) | [(dietary sugars[Title/Abstract] OR candy[Title/Abstract] OR chocolate[Title/Abstract](https://pubmed.ncbi.nlm.nih.gov/?term=%28dietary+sugars%5BTitle%2FAbstract%5D+OR+candy%5BTitle%2FAbstract%5D+OR+chocolate%5BTitle%2FAbstract%5D+OR+cacao%5BTitle%2FAbstract%5D+OR+sweeteners%5BTitle%2FAbstract%5D+OR+confection%2A%5BTitle%2FAbstract%5D%29+AND+%28%28%222011%22%5BDate+-+Publication%5D+%3A+%223000%22%5BDate+-+Publication%5D%29%29%29+AND+%28Humans%5BFilter%5D%29%29+AND+%28Review%5BPublication+Type%5D%29&sort=date&ac=no&fs=no)] OR cacao[Title/Abstract] OR sweeteners[Title/Abstract] OR confection*[Title/Abstract]) AND ("2011"[Date - Publication] : "3000"[Date - Publication]) AND Humans[Filter] AND Review[Publication Type] |
| Beverages (coffee, tea, sugar-sweetened and artificially sweetened beverages) | [575](https://pubmed.ncbi.nlm.nih.gov/?term=(coffee%5BTitle%5D%20OR%20tea%5BTitle%5D%20OR%20%22carbonated%20beverages%22%5BTitle%5D%20OR%20%22sugar-sweetened%22%5BTitle%5D%20OR%20%22soft%20drink%22%5BTitle%5D%20OR%20%22energy%20drinks%22%5BTitle%5D%20OR%20%22fruit%20juice%22%5BTitle%5D%20OR%20%22vegetable%20juice%22%5BTitle%5D%20OR%20%22non-alcoholic%20beverages%22%5BTitle%5D)%20AND%20(%222011%22%5BDate%20-%20Publication%5D%20%3A%20%223000%22%5BDate%20-%20Publication%5D)%20AND%20Humans%5BFilter%5D%20AND%20Review%5BPublication%20Type%5D&sort=pubdate&ac=no&fs=no&pos=2) (17/02/2020) | [(coffee[Title] OR tea[Title] OR "carbonated beverages"[Title] OR "sugar-sweetened"[Title] OR "soft drink"[Title] OR](https://pubmed.ncbi.nlm.nih.gov/?term=(coffee%5BTitle%5D%20OR%20tea%5BTitle%5D%20OR%20%22carbonated%20beverages%22%5BTitle%5D%20OR%20%22sugar-sweetened%22%5BTitle%5D%20OR%20%22soft%20drink%22%5BTitle%5D%20OR%20%22energy%20drinks%22%5BTitle%5D%20OR%20%22fruit%20juice%22%5BTitle%5D%20OR%20%22vegetable%20juice%22%5BTitle%5D%20OR%20%22non-alcoholic%20beverages%22%5BTitle%5D)%20AND%20(%222011%22%5BDate%20-%20Publication%5D%20%3A%20%223000%22%5BDate%20-%20Publication%5D)%20AND%20Humans%5BFilter%5D%20AND%20Review%5BPublication%20Type%5D&sort=pubdate&ac=no&fs=no&pos=2) "energy drinks"[Title] OR "fruit juice"[Title] OR "vegetable juice"[Title] OR "non-alcoholic beverages"[Title]) AND ("2011"[Date - Publication] : "3000"[Date - Publication]) AND Humans[Filter] AND Review[Publication Type] |
| Fish | [306](https://www.ncbi.nlm.nih.gov/pubmed/?term=(seafood%5BMeSH+Terms%5D)+AND+(%222011%22%5BDate+-+Publication%5D+%3A+%223000%22%5BDate+-+Publication%5D)+AND+humans%5BFilter%5D+AND+review%5BPublication+Type%5D) (27/09/2019) | [(seafood[MeSH Terms]) AND ("2011"[Date - Publication] : "3000"[Date - Publication]) AND humans[Filter] AND review[Publication Type]](https://www.ncbi.nlm.nih.gov/pubmed/?term=(seafood%5BMeSH+Terms%5D)+AND+(%222011%22%5BDate+-+Publication%5D+%3A+%223000%22%5BDate+-+Publication%5D)+AND+humans%5BFilter%5D+AND+review%5BPublication+Type%5D) |
| Food/dietary patterns | [173](https://www.ncbi.nlm.nih.gov/pubmed?term=%22diet%20quality%22%5BTitle%5D%20OR%20%22dietary%20pattern*%22%5BTitle%5D%20OR%20%22diet%20pattern*%22%5BTitle%5D%20OR%20%22eating%20pattern*%22%5BTitle%5D%20OR%20%22food%20pattern*%22%5BTitle%5D%20OR%20%22diet%20profile*%22%5BTitle%5D%20OR%20%22eating%20style*%22%5BTitle%5D%20OR%20%28DASH%5BAll%20Fields%5D%20AND%20%28%22diet%22%5BMeSH%20Terms%5D%20OR%20%22diet%22%5BAll%20Fields%5D%20OR%20%22dietary%22%5BAll%20Fields%5D%29%29%20OR%20%28%22diet%2C%20mediterranean%22%5BMeSH%20Terms%5D%20OR%20%22diet%2C%20vegetarian%22%5BMeSH%20Terms%5D%20OR%20%22diet%2C%20vegan%22%5BMeSH%20Terms%5D%29%20OR%20%28%22plant%20based%20diet%22%5BAll%20Fields%5D%20OR%20%22prudent%20diet%22%5BAll%20Fields%5D%20OR%20%22western%20diet%22%5BAll%20Fields%5D%20OR%20%22nordic%20diet%22%5BAll%20Fields%5D%20OR%20OMNIHEART%5BAll%20Fields%5D%20OR%20%22diet%20quality%20index%22%5BAll%20Fields%5D%20OR%20%22food%20score*%22%5BAll%20Fields%5D%20OR%20%22diet%20score*%22%5BAll%20Fields%5D%20OR%20%22dietary%20pattern%20score*%22%5BAll%20Fields%5D%20OR%20%22healthy%20eating%20index%22%5BAll%20Fields%5D%29%20AND%20%28%222011%22%5BPDAT%5D%20%3A%20%223000%22%5BPDAT%5D%29%20AND%20Humans%5BFilter%5D%20AND%20%28%22systematic%20review%22%5BTitle/Abstract%5D%20OR%20%22umbrella%20review%22%5BTitle/Abstract%5D%29&cmd=DetailsSearch) (30/09/2019) | ["diet quality"[Title] OR "dietary pattern*"[Title] OR "diet pattern*"[Title] OR "eating pattern*"[Title] OR "food pattern*"[Title] OR "diet profile*"[Title] OR "eating style*"[Title] OR (DASH AND dietary) OR (diet, mediterranean[MeSH Terms] OR diet, vegetarian[MeSH Terms] OR diet, vegan[MeSH Terms]) OR ("plant based diet" OR "prudent diet" OR "western diet" OR "nordic diet" OR OMNIHEART OR "diet quality index" OR "food score*" OR "diet score*" OR "dietary pattern score*" OR "healthy eating index") AND ("2011"[Date - Publication] : "3000"[Date - Publication]) AND Humans[Filter] AND ("systematic review"[Title/Abstract] OR "umbrella review"[Title/Abstract])](https://www.ncbi.nlm.nih.gov/pubmed?term=%22diet%20quality%22%5BTitle%5D%20OR%20%22dietary%20pattern*%22%5BTitle%5D%20OR%20%22diet%20pattern*%22%5BTitle%5D%20OR%20%22eating%20pattern*%22%5BTitle%5D%20OR%20%22food%20pattern*%22%5BTitle%5D%20OR%20%22diet%20profile*%22%5BTitle%5D%20OR%20%22eating%20style*%22%5BTitle%5D%20OR%20%28DASH%5BAll%20Fields%5D%20AND%20%28%22diet%22%5BMeSH%20Terms%5D%20OR%20%22diet%22%5BAll%20Fields%5D%20OR%20%22dietary%22%5BAll%20Fields%5D%29%29%20OR%20%28%22diet%2C%20mediterranean%22%5BMeSH%20Terms%5D%20OR%20%22diet%2C%20vegetarian%22%5BMeSH%20Terms%5D%20OR%20%22diet%2C%20vegan%22%5BMeSH%20Terms%5D%29%20OR%20%28%22plant%20based%20diet%22%5BAll%20Fields%5D%20OR%20%22prudent%20diet%22%5BAll%20Fields%5D%20OR%20%22western%20diet%22%5BAll%20Fields%5D%20OR%20%22nordic%20diet%22%5BAll%20Fields%5D%20OR%20OMNIHEART%5BAll%20Fields%5D%20OR%20%22diet%20quality%20index%22%5BAll%20Fields%5D%20OR%20%22food%20score*%22%5BAll%20Fields%5D%20OR%20%22diet%20score*%22%5BAll%20Fields%5D%20OR%20%22dietary%20pattern%20score*%22%5BAll%20Fields%5D%20OR%20%22healthy%20eating%20index%22%5BAll%20Fields%5D%29%20AND%20%28%222011%22%5BPDAT%5D%20%3A%20%223000%22%5BPDAT%5D%29%20AND%20Humans%5BFilter%5D%20AND%20%28%22systematic%20review%22%5BTitle/Abstract%5D%20OR%20%22umbrella%20review%22%5BTitle/Abstract%5D%29&cmd=DetailsSearch) |
| Meal pattern; intermittent fasting, fasting (meal timing) | [232](https://pubmed.ncbi.nlm.nih.gov/?term=%28%22meal+pattern*%22%5BTitle%2FAbstract%5D+OR+%22meal+frequenc*%22%5BTitle%2FAbstract%5D+OR+%22eating+frequenc*%22%5BTitle%2FAbstract%5D+OR+%22meal+tim*%22%5BTitle%2FAbstract%5D+OR+%22snacking%22%5BTitle%2FAbstract%5D+OR+%22Intermittent+fasting%22%5BTitle%2FAbstract%5D+OR+%22intermittent+energy+restriction%22%5BTitle%2FAbstract%5D+OR+%22alternate+day+fasting%22%5BTitle%2FAbstract%5D+OR+%22time+restricted+feeding%22%5BTitle%2FAbstract%5D+OR+%22meal+skipping%22%5BTitle%2FAbstract%5D%29+AND+%28%222011%22%5BDate+-+Publication%5D+%3A+%223000%22%5BDate+-+Publication%5D%29+AND+Humans%5BFilter%5D+AND+Review%5BPublication+Type%5D&sort=date) (18/02/2020) | ["meal pattern*"[Title/Abstract] OR "meal frequenc*"[Title/Abstract] OR "eating frequenc*"[Title/Abstract] OR "meal tim*"[Title/Abstract] OR "snacking"[Title/Abstract] OR "Intermittent fasting"[Title/Abstract] OR "intermittent energy restriction"[Title/Abstract] OR "alternate day fasting"[Title/Abstract] OR "time restricted feeding"[Title/Abstract] OR "meal skipping"[Title/Abstract] AND ("2011"[Date - Publication] : "3000"[Date - Publication]) AND Humans[Filter] AND Review[Publication Type]](https://pubmed.ncbi.nlm.nih.gov/?term=%22meal+pattern*%22%5BTitle%2FAbstract%5D+OR+%22meal+frequenc*%22%5BTitle%2FAbstract%5D+OR+%22eating+frequenc*%22%5BTitle%2FAbstract%5D+OR+%22meal+tim*%22%5BTitle%2FAbstract%5D+OR+%22snacking%22%5BTitle%2FAbstract%5D+OR+%22Intermittent+fasting%22%5BTitle%2FAbstract%5D+OR+%22intermittent+energy+restriction%22%5BTitle%2FAbstract%5D+OR+%22alternate+day+fasting%22%5BTitle%2FAbstract%5D+OR+%22time+restricted+feeding%22%5BTitle%2FAbstract%5D+OR+%22meal+skipping%22%5BTitle%2FAbstract%5D+AND+%28%222011%22%5BDate+-+Publication%5D+%3A+%223000%22%5BDate+-+Publication%5D%29+AND+Humans%5BFilter%5D+AND+Review%5BPublication+Type%5D) |
| Ultraprocessed foods | 228 (18/02/2020) | ["Ultra–processed food*"[Title/Abstract] OR "highly processed food*"[Title/Abstract] OR "ready-to-eat"[Title/Abstract] OR "fast food*" AND ("2011"[Date - Publication] : "3000"[Date - Publication]) AND Humans[Filter] AND Review[Publication Type]](https://pubmed.ncbi.nlm.nih.gov/?term=%22Ultra%E2%80%93processed+food%2A%22%5BTitle%2FAbstract%5D+OR+%22highly+processed+food%2A%22%5BTitle%2FAbstract%5D+OR+%22ready-to-eat%22%5BTitle%2FAbstract%5D+OR+%22fast+food%2A%22+AND+%28%222011%22%5BDate+-+Publication%5D+%3A+%223000%22%5BDate+-+Publication%5D%29+AND+Humans%5BFilter%5D+AND+Review%5BPublication+Type%5D&sort=date&ac=no&fs=no)  + brand new SR, not indexed yet: <https://www.tandfonline.com/doi/full/10.1080/09637486.2020.1725961>  Although it’s not classified as a review, this may be interesting: <https://pubmed.ncbi.nlm.nih.gov/28793996-ultra-processed-foods-in-human-health-a-critical-appraisal/> |
